# NMR-Driven Identification of Cinnamon Bud and Bark Components With Anti-Aβ Activity

**DOI:** 10.3389/fchem.2022.896253

**Published:** 2022-06-08

**Authors:** Carlotta Ciaramelli, Alessandro Palmioli, Irene Angotti, Laura Colombo, Ada De Luigi, Gessica Sala, Mario Salmona, Cristina Airoldi

**Affiliations:** ^1^ BioOrgNMR Lab, Department of Biotechnology and Biosciences, University of Milano-Bicocca, Milano, Italy; ^2^ Milan Center for Neuroscience (NeuroMI), University of Milano-Bicocca, Milano, Italy; ^3^ Department of Molecular Biochemistry and Pharmacology, Istituto di Ricerche Farmacologiche “Mario Negri”- IRCCS, Milano, Italy; ^4^ School of Medicine and Surgery, University of Milano-Bicocca, Milano, Italy

**Keywords:** Aβ peptides, Alzheimer’s disease, anti-amiloidogenic compounds, antioxidant, cinnamon, NMR metabolic profiling, UPLC-HR-MS

## Abstract

The anti-Alzheimer disease (AD) activity reported for an aqueous cinnamon bark extract prompted us to investigate and compare the anti-amyloidogenic properties of cinnamon extracts obtained from both bark and bud, the latter being a very little explored matrix. We prepared the extracts with different procedures (alcoholic, hydroalcoholic, or aqueous extractions). An efficient protocol for the rapid analysis of NMR spectra of cinnamon bud and bark extracts was set up, enabling the automatic identification and quantification of metabolites. Moreover, we exploited preparative reverse-phase (RP) chromatography to prepare fractions enriched in polyphenols, further characterized by UPLC-HR-MS. Then, we combined NMR-based molecular recognition studies, atomic force microscopy, and *in vitro* biochemical and cellular assays to investigate the anti-amyloidogenic activity of our extracts. Both bud and bark extracts showed a potent anti-amyloidogenic activity. Flavanols, particularly procyanidins, and cinnamaldehydes, are the chemical components of cinnamon hindering Aβ peptide on-pathway aggregation and toxicity in a human neuroblastoma SH-SY5Y cell line. Together with the previously reported ability to hinder tau aggregation and filament formation, these data indicate cinnamon polyphenols as natural products possessing multitarget anti-AD activity. Since cinnamon is a spice increasingly present in the human diet, our results support its use to prepare nutraceuticals useful in preventing AD through an active contrast to the biochemical processes that underlie the onset of this disease. Moreover, the structures of cinnamon components responsible for cinnamon anti-AD activities represent molecular templates for designing and synthesizing new anti-amyloidogenic drugs.

## 1 Introduction

The search for new therapeutic approaches against AD is particularly challenging as the biochemical events underlying its onset takes place several years before AD clinical manifestations (McDade and Bateman, 2017).

In this scenario, the identification of compounds, or compound mixtures, able to target, at the same time, different processes, among which oxidative stress and the on-pathway aggregation of toxic oligomers of misfolded proteins, or to induce the clearance of amyloid proteins by enhancing processes such as autophagy, can allow developing new prophylactic strategies against AD and other neurodegenerative diseases (N.D.s).

Natural extracts, being particularly rich in molecules endowed with some of the biological activities previously described, are an excellent source of new compounds with potential anti-AD activity.

When these extracts are prepared from edible sources, they also allow the production of nutraceuticals whose intake, if implemented early enough when the neuronal loss is not yet pervasive, can represent an effective preventive strategy.

Cinnamon is among the most widespread and popular spices used worldwide for cooking and in traditional and modern medicines (Rao and Gan, 2014). *Cinnamomum verum* J. Presl, (syn. *C. zeylanicum* Blume), also known as “true cinnamon,” Sri Lanka or Ceylon cinnamon, and *Cinnamomum cassia* J. Presl (syn. *C. aromaticum* Nees), also referred to as Chinese cinnamon, are the two most important cinnamon species (Kumar et al., 2019).

Among the different parts of the cinnamon tree, the most used is the bark, harvested by decorticating the branches or stems. Cinnamon bark is used as whole sticks or powder for flavoring foods, preparing tea, infusions, liqueurs, and both nonalcoholic and alcoholic drinks. Cinnamon infusions and tinctures are also widely reported in traditional medicine.

In 2011 Frydman-Marom described the potential application of cinnamon in the treatment of A.D (Frydman-Marom et al., 2011). They demonstrated that an aqueous cinnamon extract prepared from cinnamon bark reduced β-amyloid oligomerization and corrected cognitive impairment in AD animal models. Nevertheless, detailed identification of the cinnamon extract components responsible for this activity was not performed.

Here we report our effort for the molecular characterization of cinnamon inhibition of Aβ peptide aggregation and aggregates’ cytotoxicity.

To increase the chemical diversity obtainable from this plant, we prepare extracts with different extraction procedures (alcoholic, hydroalcoholic, or aqueous). Moreover, we decided to study, in addition to cinnamon bark from *C. zeylanicum* and *C. cassia*, also buds from *C. cassia* (the only one commercially available).

Cinnamon buds, the unopened flowers of the cinnamon tree that are picked just before blooming and dried in the sun, are similar to cloves in appearance. They are also used especially in the oriental culture; even if their use is quite uncommon, they are becoming widely more popular. While a vast literature on cinnamon bark biological activity is reported (Lv et al., 2017; Kumar et al., 2019), very little is known about the biological properties of cinnamon buds, and their metabolic profile is still poorly explored. The only information available concerns the chemical composition of the volatile oil obtained from buds after hydro-distillation by GC-MS analysis (Jayaprakasha et al., 2002; Kaul et al., 2003). We filled this gap by characterizing the aqueous, hydroalcoholic, and alcoholic extracts of *C. cassia* buds using NMR spectroscopy and comparing them to cinnamon barks. NMR allowed the identification of the polar metabolites in hydroalcoholic extracts and not only the volatile portion. Moreover, being an intrinsically quantitative technique, NMR provided a quantification of the metabolites contained in the extracts. Hence, preparative reversed-phase (RP) chromatography was exploited to prepare fractions enriched in polyphenols, further characterized by UPLC-HR-MS.

After their metabolic characterization, we investigated bud and bark extracts’ potential anti-amyloidogenic activity. We screened them for modulators of Aβ peptides, particularly the soluble Aβ oligomers involved in AD etiology which have the highest neurotoxicity among the other Aβ aggregates (Haass and Selkoe, 2007). We assessed the extracts’ ability to inhibit Aβ1-42 aggregation *in vitro* by ThT assay and AFM aggregate morphology studies. The extracts’ effect in the prevention of Aβ-induced neurotoxicity was tested in human SH-SY5Y neuroblastoma cells. NMR-based ligand-receptor interaction studies allowed us to identify extracts components able to bind directly soluble Aβ oligomers.

## 2 Materials and Methods

### 2.1 Cinnamon Extraction Procedures

Cinnamon samples were purchased from local retailers. The *C. zeylanicum* (CZ) bark sample sticks were purchased at the Milan Carrefour Market. A total of four packs of Carrefour brand cinnamon belonging to two different lots (L01333S and L01130S) were used. Each package contains 40 g of cinnamon sticks, with a length of about 5 cm, light hazelnut color, and a delicate scent. *C. cassia* (CC) bark sample was purchased at Flying Tiger Copenhagen store, Milan. The sample was in sticks of about 40 cm in length, dark brown, and with a pungent scent. *C. cassia* buds (BC) were purchased from “Tutte le spezie del mondo,” Anandamina S.r.l., Milan.

All the solvents and reagents used for this work were purchased from Fisher Scientific unless indicated otherwise (Fisher Scientific International inc., Pittsburgh, PA, United States).

The cinnamon barks and buds were finely ground with an electric coffee grinder, then the powders obtained were passed through a steel sieve with pores of 400 µm.

*Alcoholic extracation:* 2 g of ground cinnamon bark or buds were extracted with 20 ml of ethanol 96% v/v by sonication at 37 kHz for 60 min at 45°C in an ultrasound bath (Elmasonic P 30 H, Elma Schmidbauer GmbH, Singen, Germany). The sample was centrifuged (Eppendorf Centrifuge 5430 R, Eppendorf AG, Hamburg, Germany) at 5,752 *xg* for 10 min, at 25°C. The supernatant was filtered under *vacuum* on a Büchner funnel with Whatman 1 filter paper (11 µm–d 70 mm). The solution was concentrated under reduced pressure at 40°C (Heidolph Rotary Evaporator, Laborota 4000, Heidolph Instruments GmbH & Co. KG, Schwabach, Germany) and the solid was redissolved in MilliQ water and freeze-dried (Christ Alpha 1-2 LD plus, Martin Christ Gefriertrocknungsanlagen GmbH, Osterode am Harz, Germany). The extraction yield was calculated for each sample. Lyophilized samples were stored at −20°C.

*Hydroalcoholic extraction* (Amigoni et al., 2017): 2 g of ground cinnamon bark or buds were extracted with 20 ml of a mixture of acidified (with 0.1 M HCl) water (pH 4.5; 70%) and ethanol 96% v/v (30%) by sonication at 37 kHz for 60 min at 45°C in an ultrasound bath (Elmasonic P 30 H, Elma Schmidbauer GmbH, Singen, Germany). The sample was centrifuged (Eppendorf Centrifuge 5430 R, Eppendorf AG, Hamburg, Germany) at 5,752 *xg* for 10 min, at 25°C. The supernatant was filtered under *vacuum* on a Büchner funnel with Whatman 1 filter paper (11 µm–d 70 mm). The solution was concentrated under reduced pressure at 40°C to remove ethanol (Heidolph Rotary Evaporator, Laborota 4,000, Heidolph Instruments GmbH & Co. KG, Schwabach, Germany) and the aqueous solution was freeze-dried (Christ Alpha 1-2 LD plus, Martin Christ Gefriertrocknungsanlagen GmbH, Osterode am Harz, Germany). The extraction yield was calculated for each sample. Lyophilized samples were stored at −20°C.

*Aqueous extraction:* 2 g of ground cinnamon bark or buds were extracted with 20 ml of acidified (with 0.1 M HCl) water (pH 4.5) by sonication at 37 kHz for 60 min at 45°C in an ultrasound bath (Elmasonic P 30 H, Elma Schmidbauer GmbH, Singen, Germany). The sample was centrifuged (Eppendorf Centrifuge 5430 R, Eppendorf AG, Hamburg, Germany) at 5,752 *xg* for 10 min, 25°C. The supernatant was filtered under *vacuum* on a Büchner funnel with Whatman 1 filter paper (11 µm–d 70 mm) and the solution was freeze-dried (Christ Alpha 1-2 LD plus, Martin Christ Gefriertrocknungsanlagen GmbH, Osterode am Harz, Germany). The extraction yield was calculated for each sample. Lyophilized samples were stored at −20°C.

Samples were extracted with each of the different procedures and analyzed in triplicate.

### 2.2 NMR Metabolic Profiling of Extracts

Freeze-dried samples of cinnamon extracts were suspended in D_2_O or CD_3_OD at a final concentration of 15 mg/ml. Samples were sonicated (37 kHz, 20 min, Elmasonic P 30 H, Elma Schmidbauer GmbH, Singen, Germany) and centrifuged (9,425 *xg* for 15 min, 20°C, ScanSpeed 1730R Labogene, Lynge, Sweden) before NMR analyses. The 4,4-Dimethyl-4-silapentane-1-sulfonic acid (DSS) was added to the sample at the final concentration of 1 mM as an internal reference for concentrations and chemical shifts. The pH of samples dissolved in D_2_O was verified with a microelectrode (InLab Micro electrode and Five Easy pHmeter, Mettler Toledo, Columbus, OH, United States) and adjusted to 7.2 with NaOD or DCl addition and corrected for the isotope effect.

NMR experiments were performed at 25°C. All spectra were acquired on a Bruker AVANCE III 600 MHz NMR spectrometer (Bruker, Billerica, MA, United States), equipped with a QCI (^1^H, ^13^C, ^15^N/^31^P, and 2H lock) cryogenic probe. ^1^H-NMR spectra were recorded with *zg* (^1^H in CD_3_OD) and *noesygppr1d*, *cpmgpr1d,* and *ledbpgppr2s1d* (^1^H in D_2_O) pulse sequences the in Bruker library and 256 scans, spectral width 20 ppm, and a relaxation delay of 5 s. They were processed with 0.3 Hz line broadening, automatically phased and baseline corrected. Chemical shifts were internally calibrated to the DSS peak at 0.00 ppm. The ^1^H,^1^H-TOCSY (Total Correlation SpectroscopY) spectra were acquired with 32 scans and 512 increments, 80 ms mixing time, and a relaxation delay of 2 s ^1^H,^13^C-HSQC (Heteronuclear Single Quantum Coherence) spectra were acquired with 48 scans and 512 increments, relaxation delay 2 s. MestReNova software package of Mestrelab (MestReNova v 14.2.1-27684, 2021, Mestrelab Research, Santiago de Compostela, Spain) was used for NMR spectra processing and peak picking.

The assignment of resonances and the identification of compounds were done with the support of 2D NMR spectra, libraries of our laboratory (Airoldi et al., 2018a; Airoldi et al., 2019; Airoldi et al., 2020), the online databases *Human Metabolome Database* (HMDB, http://www.hmdb.ca), *Biological Magnetic Resonance Data Bank* (BMRB, http://www.bmrb.wisc.edu), *FooDB* (https://foodb.ca), *Birmingham Metabolite Library* (http://www.bml-nmr.org), and compared with reported assignments (Lei et al., 2015; Farag et al., 2018).

The MNova GSD (Global Spectrum Deconvolution) algorithm was employed to deconvolute the overlapping regions, allowing the absolute quantification of metabolites with resonances in crowded spectral areas. The Simple Mixture Analysis (SMA) tool (Simple Mixture Analysis (SMA) version 2.0, mestrelab.com/software/mnova/sma/) integrated in the MestreNova software package of Mestrelab was used to set a semiautomatic protocol for the identification and quantification of metabolites. Specific libraries for the matrix of interest were built and used in this work (Airoldi et al., 2021). When possible, the concentration of the compound was calculated as the mean value of the different assigned signals (Airoldi et al., 2016). Metabolite concentrations were reported as means (M) ± standard deviations (SD) of triplicate experiments.

### 2.3 Determination of Antioxidant Activity

The antioxidant activity was determined through three different spectrophotometric assays (Folin-Ciocalteu, ABTS, and DPPH assay) as previously reported (Amigoni et al., 2017; Ciaramelli et al., 2018; Palmioli et al., 2019) and briefly detailed below. Absorbance measurements were performed with Varian Cary 50 Scan UV–Visible Spectrophotometer (Agilent, Santa Clara, CA, United States) using disposable polymethyl methacrylate (PMMA) semimicro 10 mm-cuvettes relative to a blank solution.

Folin–Ciocalteu’s method assay determined total reducing capacity (or total polyphenolic content). Briefly, 80 μl of diluted samples (or standards/blank) and 40 μl of Folin’s reagent were dispensed in a cuvette containing 400 μl of H_2_O. Then, 480 μl of Na_2_CO_3_ 10.75% (w/v) solution was added and after 30 min of incubation at room temperature, absorbance was read at 760 nm. Samples were diluted to 0.2 mg/ml and standard solutions of gallic acid (0–100 μg/ml) were used for calibration (linear fitting R^2^ > 0.98, *n* = 5). Results were expressed as mg of gallic acid equivalent (GAE) on g of extract. Data were reported as means (±SD) of triplicate measurements.

Radical scavenging was determined by ABTS and DPPH method assays. ABTS assay is based on the scavenging ability of antioxidants to the long-life intense colored radical cation 2,2′-azino-bis(3-ethylbenzothiazoline-6-sulphonic acid. A 7 mM stock solution of ABTS^·+^ was produced by mixing an equal amount of a 14 mM ABTS solution and a 4.9 mM K_2_S_2_O_8_ solution in H_2_O (final concentration 7.00 mM and 2.45 mM, respectively). The mixture was left at room temperature in the dark for at least 12–16 h before use and stored at 4°C for 7 days. A working solution of ABTS^·+^ was prepared daily by diluting the stock solution (1:50, abs 0.70 ± 0.05 at 734 nm). Briefly, 50 μl of the sample (or standards) were added in a cuvette containing 950 μl of ABTS^·+^ solution, and the absorbance at 734 nm was read after 30 min of incubation at room temperature. DPPH assay is based on the scavenging of the stable free-radical 2,2-diphenyl-1-picrylhydrazyl. Briefly, 950 μl of a diluted solution of DPPH in buffered MeOH (100 μM in a mixture of 60% MeOH and 40% acetate buffer pH 4.5, Abs 0.70 ± 0.05) and 50 μl of a diluted sample (or standard) were added into a cuvette, the absorbance at 517 nm was read after 30 min of incubation at room temperature. Samples were diluted to 0.2 mg/ml and standard solutions of Trolox (6-hydroxy-2,5,7,8-tetramethylchroman-2-carboxylic acid, a water-soluble analogue of vitamin E) were used for calibration (0–500 μM, linear fitting R^2^ > 0.98, *n* = 7). The results were expressed as mg of Trolox equivalent (TE) on g of extract And the data were reported as means (±SD) of triplicate measurements.

### 2.4 Preparative Reverse Phase C18 Column Chromatography

Automated flash chromatography was done on a Biotage^®^ Isolera™ Prime system (Biotage AB, Uppsala, Sweden) equipped with a Spektra package. A solution of the extracted sample (200 mg in 5 ml of H_2_O) was loaded into a SNAP KP-C18-HS (12 g) cartridge, equipped with a precolumn Biotage’s Samplet^®^ cartridge SNAP-C18 (1 g). Column chromatography was performed using water (A) and methanol (B) as eluent solvents. A linear elution gradient was applied (2% B for 2 CV, 2%–100% of B in 15 CV, and 100% B for 3 CV) at 12 ml/min flow rate. The eluate was automatically collected in fractions based on photodiode array detector signal (range 200–400 nm) and UV detection at λ = 280 nm and λ = 320 nm. Fractions were then pooled in homogenous groups, the organic solvent was removed under reduced pressure and residues were freeze-dried (Christ ALPHA 1-2 LD PLUS, Martin Christ Gefriertrocknungsanlagen GmbH, Osterode am Harz, Germany), obtaining fractions A-D.

### 2.5 Ultra-Performance Liquid Chromatography Coupled With Electrospray Ionization-High Resolution Mass Spectrometry (UPLC/ESI-HR-MS)

The UPLC/ESI-HR-MS analysis was carried out by a Waters^®^ Acquity™ ultra-performance liquid chromatography (UPLC) system consisting of a quaternary solvent manager and a sample manager coupled with an in-line Waters photodiode array (PDA) detector, Waters Xevo G2-XS quadrupole time-of-flight (QTof) mass spectrometer and an analytical workstation with Waters MassLynx™ 4.2 software (Waters, Milford, MA, United States). Separations were carried out with a Waters^®^ Acquity™ Premier HSS T3 column [100 × 2.1 mm I.D., 1.8 μm) coupled with a VanGuard™ HSS T3 guard column. The mobile phase consisted of water (A) and acetonitrile (B)], both modified with 0.1% formic acid. The samples were dissolved in 10% aqueous acetonitrile at the 1 mg/ml concentration and 2 μl were injected into the column. The eluting conditions were isocratic 5% B (0–1 min), linear gradient from 5% to 50% B (1–11 min), 50%–90% B (11–12 min), then the column was washed with 90% B (12–15 min), and then back to 5% B in (15–16 min) and equilibrate at 5% B for 4 min (16–20 min) before the next run. The flow rate was 0.4 ml/min, and the column temperature was maintained at 40°C. Mass detection was performed with an electrospray ionization (ESI) source operating in positive (ES+) and negative (ES-) ion mode and in sensitivity mode. The capillary voltage and the cone voltage were set to +3/-2 kV and 40 V. The source temperature was set to 120°C and the desolvation gas flow was set to 1000 L/h at a temperature of 350°C with the cone gas set to 50 L/h. Mass calibration was performed with Sodium Formate calibration solution and a Leucine Enkephalin (LeuEnk, m/z 556.2771 in positive and 554.2615 in negative ion mode) was used as lock mass for accurate mass calibration. The lock mass solution was injected through the LockSpray interface at 10 µl/min every 30 s with a reference capillary voltage of +3/−2 kV and at the concentration was 100 pg/µl in 50% aqueous acetonitrile, 0.1% formic acid.

Data were acquired by a Full MS scan and data-dependent tandem MS analysis of the five most intense ions (Top 5) over a mass range of 50–1,200 m/z (FastDDA experiment). The full-scan survey was applied to trigger MS/MS acquisition of precursor ions with a threshold higher than 5,000 intensity per second and switch back to a full-scan survey after 5 MS/MS scan. Full scan spectra were acquired at a scan time of 0.2 s, and MS/MS spectra acquisition at a scan time of 0.1 s. The dynamic collision energy was set to 6–9 V for 50 Da and 60–80 V for 1,200 Da.

The Full MS scan data were processed using MestreNova 14.2.3 (Mnova MS plug-in, Mestrelab Research, Santiago de Compostela, Spain). The DDA raw data were processed using MS-Dial 4.80 (http://prime.psc.riken.jp/compms/msdial/main.html) for ion deconvolution and peak alignment. The tolerances of retention time, MS1, and MS2 were set at 0.01 min, 0.02 Da, and 0.05 Da, respectively. The minimum peak height and MS/MS abundance threshold were set at 1,000 and 10 amplitudes, respectively. Sigma window value was 0.5, and mass slice width was 0.1 Da.

Compound identification was performed according to their calculated accurate mass, isotopic patterns, and structures were confirmed by comparing MS/MS spectra with those reported in the literature or public databases (HMDB https://hmdb.ca/, GNPS https://gnps.ucsd.edu/,and MassBank https://massbank.eu/MassBank/).

### 2.6 Peptide Synthesis

Synthetic Aβ1-42 (DAEFRHDSGYEVHHQKLVFFAEDVGSNKGAIIGLMVGGVVIA) peptide was prepared on a Syro I synthesizer (Biotage, Uppsala, Sweden) using Fmoc-protected L-amino acid derivatives, NOVASYN-TGA resin and a 0.1 mM scale. The peptide was cleaved from the resin as previously described (Manzoni et al., 2009) and purified by reverse-phase HPLC on a semipreparative C4 column (Waters) using water: acetonitrile gradient elution. Peptide identity was confirmed by MALDI-TOF analyses (model Reflex III, Bruker). The purity of peptides was always above 95%.

### 2.7 Atomic Force Microscopy (AFM)

Aβ1-42 was dissolved as previously described to 2.5 μM with or without cinnamon extracts or the fractions (2.5 μg/ml) and was incubated in quiescent conditions at 37°C for 24 h. After incubation, 30 μl of samples were spotted onto a freshly cleaved Muscovite mica disk and incubated for 7 min. The excess of the sample on the mica disk was washed with 10 ml MilliQ water and dried under a gentle nitrogen stream. Samples were mounted onto a Multimode AFM with a NanoScope V system (Veeco/Digital Instruments) operating in Tapping Mode and measurements were carried out using 0.01–0.025 Ω/cm antimony-doped silicon probes (T: 3.5–4.5 μm, L: 115–135 μm, W: 30–40 μm, k: 20–80 N/m, f0: 323–380 kHz, Bruker AFM probes) with a scan rate in the 0.5–1.2 Hz range, proportional to the area scanned. Measurements confirmed all the topographic patterns in a minimum of four different separated areas, and in order to exclude the interference of possible artifacts, freshly cleaved mica soaked with 30 µl of PB 50 mM was also analyzed as controls. Samples were analyzed with the Scanning Probe Image Processor (SPIP Version 5.1.6 released 13 April 2011) data analysis package.

### 2.8 Thioflavin T Binding Assay

Aβ1-42 were dissolved in 10 mM NaOH, H_2_O, and PB 50 mM (1:1:2) to 2.5 μM in the absence or presence of the cinnamon extracts or the fractions (2.5 μg/ml) and were incubated at 37°C in 20 µM ThT (Sigma) in 96 well black plates (Isoplate, Perkin Elmer). A plate reader monitored the ThT fluorescence for 24 h by a plate reader (Infinite F500 Tecan: excitation 448 nm, emission 485 nm, 37°C). Data were expressed as the mean of three replicates, calculated by subtracting the relative control solutions (extracts or fractions alone), and expressed as the percentage of reduction of Aβ1-42 aggregation.

### 2.9 NMR Binding Studies

To obtain samples containing Aβ oligomers, lyophilized Aβ1-42 was dissolved in 10 mM NaOD, then diluted 1:1 with 20 mM phosphate buffer (pH 7.4) and cinnamon extracts (5 mg/ml) or enriched fractions (5 mg/ml). The pH of each sample was measured with a Microelectrode (InLab Micro, Mettler Toledo, Columbus, OH, United States) and adjusted to pH 7.4 with NaOD and/or DCl. All pH values were corrected for the isotope effect. Experiments were run on an AVANCE III 600 MHz NMR spectrometer (Bruker, Billerica, MA, United States) equipped with a QCI (^1^H, ^13^C, ^15^N/^31^P, and ^2^H lock) cryogenic probe.

A basic sequence from the Bruker library was used for the STD experiments. A train of Gaussian-shaped pulses of 50 ms each was employed to saturate the protein envelope selectively; the total saturation time of the protein envelope was adjusted to the number of shaped pulses and set at 2 s. On- and off-resonance spectra were acquired in an interleaved mode with the same number of scans. The STD NMR spectrum was obtained by subtracting the on-resonance spectrum from the off-resonance spectrum.

### 2.10 *In Vitro* Toxicity Assay

Human neuroblastoma SH-SY5Y cell line was grown in Dulbecco’s Modified Eagle’s medium (DMEM, Gibco) supplemented with l-glutamine (2 mM, Gibco), antibiotics (penicillin/streptomycin 10,000 U, Lonza) and 10% heat-inactivated foetal calf serum (FCS, Gibco). SH-SY5Y cell line was seeded in 96 wells plate (10^5^ cell/ml) and overnight incubated (37°C, in humidified 5% CO_2_ atmosphere). After completing planting the medium was changed with 1% of FCS in DMEM, to reduce cell growth.

Aβ1-42 was dissolved in 10 mM NaOH, H_2_O and PBS (1:1:2) and added to the cinnamon extracts or the fractions (μg/ml), before the treatment of SH-SY5Y cell line, to obtain the final concentration of 10 μM for Aβ1-42 in the well. The cytotoxicity was evaluated using the MTT reduction assay after 24 h of incubation. Tetrazolium solution (20 μl of 5 mg/ml, Sigma Aldrich) was added to each well and incubated for 4 h. The medium was replaced with acidified isopropanol (0.04 M HCl) to dissolve the purple precipitate and the absorbance intensity was measured at 570 nm using a plate reader (Infinite M200, Tecan). The data were expressed as a percentage compared to controls (solvent) of three different replicates.

### 2.11 Assessment of Autophagy Markers

Gene and protein expression of autophagy markers were assessed by real-time quantitative PCR (qPCR) and Western blot, respectively.

For qPCR assays, total RNA was extracted using the RNeasy Mini kit (Qiagen), retrotranscribed (2 µg) into cDNA using the SuperScript VILO cDNA Synthesis Kit (Invitrogen) and amplified (50 ng for LC3, beclin-1, p62, Hsc70 ,and BDNF and 100 ng for Lamp2A) in triplicate in the ABI Prism 7,500 HTSequence Detection System (Applied Biosystems), at the conditions already published (Sala et al., 2021). For relative quantification of each target vs. β-actin mRNA, the comparative C_T_ method was used. The sequences of the primers used (Sigma-Aldrich) are the following:

**Table:** 

LC3	F	CAG​CAT​CCA​ACC​AAA​ATC​CC
	R	GTT​GAC​ATG​GTC​AGG​TAC​AAG
beclin-1	F	ATC​TCG​AGA​AGG​TCC​AGG​CT
	R	CTG​TCC​ACT​GTG​CCA​GAT​GT
p62	F	CCA​GAG​AGT​TCC​AGC​ACA​GA
	R	CCG​ACT​CCA​TCT​GTT​CCT​CA
Lamp2A	F	GCA​GTG​CAG​ATG​AAG​ACA​AC
	R	AGT​ATG​ATG​GCG​CTT​GAG​AC
Hsc70	F	CAG​GTT​TAT​GAA​GGC​GAG​CGT​GCC
	R	GGG​TGC​AGG​AGG​TAT​GCC​TGT​GA
BDNF	F	TGG​CTG​ACA​CTT​TCG​AAC​AC
	R	AGA​AGA​GGA​GGC​TCC​AAA​GG
β-actin	F	TGT​GGC​ATC​CAC​GAA​ACT​AC
	R	GGA​GCA​ATG​ATC​TTG​ATC​TTC​A

For Western blot analysis, cell pellets were lysed in cell extraction buffer (Invitrogen) supplemented with 1 mM PMSF (Sigma-Aldrich) and protease inhibitor cocktail (Sigma-Aldrich). Protein concentration was assessed by Bradford’s method and sample lysates diluted in Laemmli’s loading buffer pH 6.8. After denaturation at 95°C for 4 min, samples were separated by SDS-PAGE in 4%–12% tris glycine gels (Invitrogen) and transferred to nitrocellulose. Blots were blocked for 1 h, incubated overnight at 4°C with specific primary antibodies (beclin-1, Cell Signaling, 1:1000 dilution; p62, Cell Signaling, 1:1000 dilution, Lamp2A, Abcam, 1:900 dilution) and then with HRP-linked anti-mouse or -rabbit IgG antibody (Sigma-Aldrich) for 1 h. Chemiluminescence revealed signals, detected using the ImageQuant 800 (Amersham) imaging system and quantified using the ImageJ software. Protein expression was calculated as the ratio between optical densities of the target protein and internal standard (β-actin) and expressed as a percentage vs. the vehicle-treated cells.

### 2.12 Statistical Analysis

Data were reported as mean with standard deviation and compared with one-way variance analysis, followed by the appropriate posthoc test (GraphPad Prism Software).

## 3 Results

### 3.1 Cinnamon Extracts’ NMR Metabolic Profiling

Cinnamon extracts were prepared from three different matrices: *C. cassia* buds (BC), *C. cassia* bark (CC) and *C. zeylanicum* bark (CZ). Ultrasound-assisted extractions were performed using different solvents: alcoholic extraction in ethanol (E), hydroalcoholic extraction in acidified MilliQ water (pH 4.5) and ethanol in ratio 7:3 (HE), aqueous extraction with acidified (pH 4.5) MilliQ water (H). The extraction procedures were performed at least in triplicate. As already mentioned, the extraction procedures were chosen to mimic those used in cooking and traditional medicine as much as possible.

The metabolic profile of extracts was characterized by NMR spectroscopy. For the preparation of NMR samples, two different solvents were chosen. All the samples were dissolved in CD_3_OD, as all the extracts showed good solubility in this solvent. Due to the poor solubility of the alcoholic and hydroalcoholic extracts in water, only samples from ultrasound-assisted aqueous extraction were dissolved in D_2_O.

**FIGURE 1:**
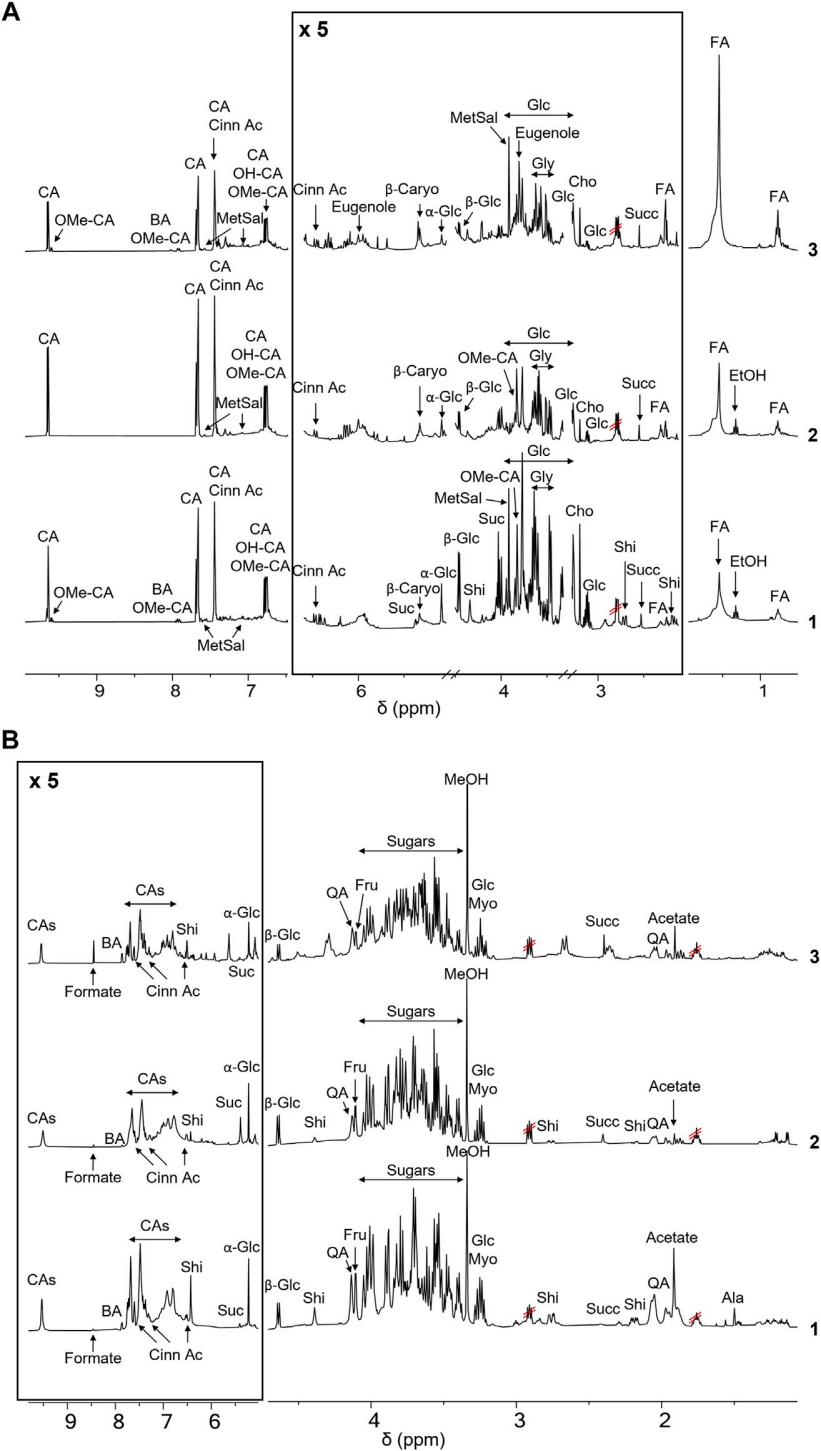
NMR-based metabolic profiling of cinnamon extracts. **(A)** ^1^H-NMR spectra of the ethanol extracts of cinnamon buds **1**) and bark **2**, *C. cassia*; **3**) *C. zeylanicum*) samples. Each sample containing 15 mg/ml of extract, was dissolved in CD_3_OD, DSS 1 mM. Spectra were acquired at 25°C and 600 MHz. Assignments of the resonances of the most important metabolites are reported (CA (E)-Cinnamaldehyde; OMe-CA, 2-Methoxycinnamaldehyde; BA, Benzoic Acid; Cinn Ac, Cinnamic Acid; OH-CA, 2-Hydroxycinnamaldehyde; MetSal, Methyl Salicylate; β-Caryo, β-Caryophyllene; Suc, Sucrose; Glc, Glucose; Shi, Shikimic Acid; Gly, Glycerol; Cho, Choline; Succ, Succinate; FA, Fatty Acid). **(B)** ^1^H-NMR spectra of the aqueous extracts of buds (**1**) and bark (2, C. cassia; 3, C. *zeylanicum*) samples. Each sample, containing 15 mg/ml of extract, was dissolved in D_2_O, pH 7.2, and DSS 1 mM. Spectra were acquired at 25°C and 600 MHz. Assignments of the resonances of the most important metabolites are reported (CAs (E)-Cinnamaldehyde and derivatives; BA, Benzoic Acid; Cinn Ac, Cinnamic Acid; Suc, Sucrose; Glc, Glucose; Shi, Shikimic Acid; QA, Quinic Acid; Fru, Fructose; Myo, *Myo*-inositol; Succ, Succinate).

This approach allowed the comparison of the metabolic profiles of buds from *C. cassia* and bark cinnamon extracts from *C. cassia* and *C. zeyalanicum*, obtained with different extraction solvents and acquired both in CD_3_OD and D_2_O (Figure 1; Table 1). Representative NMR spectra of cinnamon extracts dissolved in CD_3_OD and D_2_O are depicted in Figure 1A (and in Supplementary Figure S1) and Figure 1B, respectively, and the assignments of ^1^H and ^13^C resonances are reported in Table 1.

**TABLE 1:** Assignments of ^1^H and13C chemical shifts of the resonances of metabolites contained in cinnamon extracts as found in ^1^H-NMR, ^1^H,^1^H-TOCSY, and ^1^H,^13^C-HSQC spectra of samples dissolved in D_2_O and CD_3_OD. ND (not detected) indicates that the resonances of the metabolites were not found in the ^1^H-NMR spectrum of the sample. Missing values of chemical shifts are due to overlapped resonances or signals with too low intensity.

	Metabolite	D_2_O			CD_3_OD		
		Assignment	^1^H chemical shift (ppm)	13C chemical shift (ppm)	Assignment	^1^H chemical shift (ppm)	^13^C chemical shift (ppm)
1	Acetate	CH_3_	1,91(s)	26,13	CH_3_	1,91–1,94 (s)	22,18
2	Alanine	CHα	3,77 (m)		ND		
		CH_3_	1,47 (d)				
3	Benzoic acid	CH (3,5)	7,48 (m)	132,67	CH (3;5)	7,42 (m)	
		CH (4)	7,54 (m)	121,76	CH (4)	7,51 (m)	
		CH (2,6)	7,86 (d)		CH (2;6)	7,98 (d)	131,92
4	2,3-Butanediol	2 x CH_3_	1,13 (d, J= 5.9 Hz)		2 x CH_3_	1,13 (d)	
		CH (1)	3,62 (m)				
		CH (2)	3,72 (m)				
5	Capric acid	CH (12)	0,86 (d)		ND		
		CH (6,7,8,9,10,11)	1,29 (m)				
		CH (5)	1,53 (d)				
		CH (4)	2,16 (m)				
6	β-Caryophyllene	ND			CH (5)	5,33 (m)	132,28
					CH_2_ (3,6,7)	2,01–1,96 (m)	
					CH_2_ (2)	1,31 (m)	
7	Choline	3 x CH_3_	3,21 (s)	56,17	3 x CH_3_	3,20 (s)	55,25
					CH_2_ (2)	3,47 (m)	68,36
8	(E)-Cinnamaldehyde	CHO (1)	9,51 (m)	200,50	CHO (1)	9,65 (d)	197,67
		CH (5,9)	7,66 (m)	131,50	CH (5,9)	7,67 (m)	131,31
		CH (3)	7,66 (m)	158,80	CH (3)	7,67 (m)	156,90
		CH (7)	7,45 (m)	131,60	CH (7)	7,44 (m)	131,75
		CH (6,8)	7,45 (m)	134,30	CH (6,8)	7,44 (m)	133,72
		CH (2)	6,79 (m)	130,00	CH (2)	6,77 (dd)	131,08
9	(Z)-Cinnamaldehyde	ND			CHO (1)	9,79 (d, J = 7,8 Hz)	
					CH (3)	7,7 (m)	
					CH (2)	6,78 (dd)	
10	Cinnamaldehyde dimethylacetal	ND			CH (3)	4,92 (d, J = 5,1 Hz)	106,36
					CH (2)	6,14 (dd, J = 16,2–5,2 Hz)	128,23
					CH (1)	6,71 (d, J = 16,0 Hz)	136,57
11	Cinnamic acid	CH (8)	6,52 (d)	126,87	CH (8)	6,48 (d, J = 16,0 Hz)	120,90
		CH (7)	7,39 (d)	143,57	CH (7)		
		CH (3,5)	7,43 (m)	131,51	CH (3,4,5)		
		CH (4)	7,43 (m)	131,82			
		CH (2,6)	7,61 (d)	130,44	CH (2,6)	7,69 (d)	147,63
12	Coumarin	ND			CH (3)	8,02 (d, J = 7,1 Hz)	132,23
					CH (6,9)	7,59 (m)	136,10
					CH (7,8)	7,47 (m)	131,22
13	Ethanol^a^	ND			CH_2_	3,61 (q)	59,65
					CH_3_	1,16 (t)	20,02
14	Eugenol	ND			CH (3,6)	6,71 (m)	115,00
					CH (5)	6,62 (m)	123,67
					CH (8)	5,93 (m)	141,14
					CH_2_ (9)	5,03 - 5,00 (ddt)	117,22
					OMe	3,80 (s)	58,14
					CH_2_ (7)	3,28 (m)	42,78
15	Fatty acid (FA)	CH_3_ (ω0)	0,85 (t)		CH_3_ (ω0)	0,89 (t, J = 6,9 Hz)	16,52
		CH_2_ (ω1)			CH_2_ (ω1)	1,32 (m)	25,82
		CH_2_ (ω2)	1,28 (m)		CH_2_ (ω2)	1,28 (m)	32,34
		CH_2_ (3)	1,53 (m)		CH_2_ (3)	1,59 (m)	27,77
		CH_2_ (4)	2,16 (m)		CH_2_ (4)	2,04 (m)	30,11
		CH_2_ (2)			CH_2_ (2)	2,26 (t, J = 7,4 Hz)	36,87
16	Formate	CH (1)	8,45 (s)		CH (1)	8,44 (s)	
17	Fructose (furanose)	CH (4)	4,11 (m)	73,50	ND		
		CH (3,5)	3,81 (m)	66,60			
		CH (6.1 b)	3,67 (m)	65,00			
18	GABA (γ-Aminobutyric acid)	CH (2)	1.89 (m)		ND		
		CH (3)	2.30 (m)				
		CH (1)	3.00 (m)	38.76			
19	α-Galactose	CH (1)	5,25 (d)	94,57	CH (1)	5,14 (d, J = 2,4 Hz)	
		CH (2)	3,78 (m)		CH (2)	3,55 (m)	
		CH (3)	3,85 (m)	72,06	CH (3)	3,63 (m)	
		CH (4)	3,97 (m)		CH (4)	3,80 (m)	
		CH (5)	4,07 (m)	73,35			
	β-Galactose	CH (1)	4,58 (d)	98,88			
20	α-Glucose	CH (1)	5,22 (d)	94,47	CH (1)	5,09 (d, J = 3,7 Hz)	95,35
		CH (2)	3,83 (m)	73,90	CH (2)	3,9 (m)	75,06
		CH2 (6)	3,82–3,76 (m)	65,00	CH2 (6)	3,83–3,77 (m)	64,20
		CH (3)	3,7 (m)	75,25	CH (3)	3,77 (m)	
		CH (5)	3,52 (m)	73,66	CH (5)	3,66 (m)	76,29
		CH (4)	3,40 (m)	72,04	CH (4)	3,34 (m)	75,00
	β-Glucose	CH (1)	4,63 (d)	98,31	CH (1)	4,46 (d, J = 7,8 Hz)	99,57
		CH2 (6)	3,71 - 3,89 (m)	63,21	CH_2_ (6)	4,00–3,83 (dd)	65,95
		CH (3), CH (5)	3,48 (m)	78,20	CH (3), CH (5)	3,45–3,34	78 - 75
		CH (4)	3,40 (m)	72,10	CH (4)	3,27	73,19
		CH (2)	3,23 (m)	76,59	CH (2)	3,11	76,68
21	Glycerol	CH_2_ (1,3)	3,65 (dd) - 3,56 (dd)	65,00	CH_2_ (1,3)	3,59 (dd)-3,52 (dd)	65,79
22	*2*-Hydroxycinnamaldehyde	ND			CHO (1)	9,50 (d, J = 7,8 Hz)	
					CH (3,5,9)	7,66 (m)	
					CH (2)	6,76 (m)	
23	Malic acid	CH_2_ (3)	2,36 (dd) - 2,67 (d)	45,40	ND		
		CH (2)	4,30 (d)	73,24			
24	Methanol^b^	CH_3_	3,34 (s)	49,90	CH_3_	3,29 (s)	50,78
25	2-Methoxycinnamaldehyde	ND			CHO (1)	9,60 (d, J = 8,0 Hz)	198,16
					CH (3)	7,92 (d, J = 16,0 Hz)	152,40
					CH (2)	6,82 (m)	131,60
					OMe	3,83 (s)	57,83
26	Methyl salicylate	ND			CH (7)	7,62 (dd, *J* =7,7–1,7 Hz)	132,11
					CH (4)	7,07 (d, J = 8,4 Hz)	114,41
					CH (6)	7,02 (m)	117,46
					CH (5)	7,45 (m)	134,12
					OMe	3,91 (s)	57,90
27	*Myo*-inositol	CH (5)	3,27 (t)	77,13	ND		
		CH (4,6)	3,51 (d)	73,98			
		CH (1,3)	3,61 (m)	75,15			
		CH (2)	4,05 (t)	74,93			
28	Phenylalanine	ND			CH (7,9)	7,41 (d)	129,46
					CH (8)	7,31 (t, J = 7,7 Hz)	131,41
					CH (6,10)	7,24 (t, J = 7,2 Hz)	131,06
					CHα	3,86 (m)	
					CH_2_	3,13–3,26 (m)	
29	Quinic acid	CH (7)	1.86 (m)	43,47	ND		
		CH (6)	1.96 (dt)	40,21			
		CH (5)	2.05 (m)	40,15			
		CH (4)	2.05 (m)	43,47			
		CH (3)	3.55 (m)	77.95			
		CH (2)	4.00 (m)	69.73			
		CH (1)	4.13 (dd)	73,19			
30	Shikimic acid	CH (2)	6.43 (m)	133,83	CH (2)	6,74 (m)	117,50
		CH (3)	4.39 (t)	68,70	CH (3)	4,34 (t)	68,85
		CH (5)	3.98 (m)	69,52	CH (5)	3,96 (m)	69,80
		CH (4)	3.70 (m)	74.81	CH (4)	3,64 (m)	76,24
		CH_2_ (6)	2,76 (dd) - 2,19 (dd)	35,23	CH_2_ (6)	2,7 (dd) - 2,18 (dd)	33,70
31	Succinate	2 x CH_2_	2,40 (s)	36,46	2 x CH_2_	2,55 (s)	31,64
32	Sucrose	CH (7) (Glc)	5,40 (d)	101,99	CH (7) (Glc)	5,38 (d)	94,99
		CH (3) (Fru)	4,21 (d)	79,21	CH (3) (Fru)	4,16 (d)	68,85
		CH (4) (Fru)	4,04 (t)	74,60	CH (4) (Fru)		
		CH (5) (Fru)	3,96 (m)	84,18	CH (5) (Fru)	3,94 (m)	
		CH (9) (Glc)	3,83 (m)	74,94	CH (9) (Glc)	3,80 (m)	
		CH_2_ (17 + 19)	3,81 (m)	63,05–64,90	CH_2_ (17 + 19)		
		CH (11) (Glc)	3,75 (t)	73,75	CH (11) (Glc)	3,68 (t)	
		CH_2_ (13) (Fru)	3,63 (s)	65,00	CH_2_ (13) (Fru)		
		CH (12) (Glc)	3,56 (dd)	73,77	CH (12) (Glc)		
		CH (10) (Glc)	3,42 (t)	72,12	CH (10) (Glc)	3,40 (t)	
33	Cinnamaldehyde derivative #1	ND				9,91 (d)	
						7,72 (m)	
34	Cinnamaldehyde derivative #2	ND				9,59 (d)	198,55
						7,90 (d, J= 16,0 Hz)	153,33
						6,86 (m)	131,80
35	Cinnamaldehyde derivative #3	ND				9,56 (d, J= 7.8 Hz)	
						7,58 (m)	
						6,64 (m)	
36	Unknown A	ND				8,03 (m)	
						7,59 (m)	
						7,48 (m)	
						6,34 (m)	
37	Unknown B	ND				7,54 (m)	
						7,26 (m)	
						6,88 (m)	
38	Unknown C	ND				5,35 (s)	69,51
						1,95 (m)	
						1,53 (m)	
39	Unknown D	ND				5,25 (m)	
						4,34 (m)	
						4,16 (m)	
40	Unknown E	ND			CH_2_	4,70 (dd, J = 6,4–1,5 Hz)	67,87
						6,67 (m)	
						6,32 (m)	
41	Unknown F	ND				4,21 (dd, J = 5,7–1,6 Hz)	65,52
						6,60 (m)	
						6,31 (m)	
42	Unknown G	ND			CH_2_	4,16 (m)	31,50
						2,70 (m)	
43	Unknown H	ND				4.01 (dd, J = 12,3–1,1 Hz)	
						3,83 (m)	
						3,61 (m)	
44	Unknown I	ND			CH_2_	3,62 (d)	66,57
						2,67 (m)	
45	Unknown J		1,21 (d, J= 6.5 Hz)		ND		
			3,41 (m)				
			3,91 (m)				

a Extraction solvent for HE and E extracts.

b NMR solvent for samples in CD_3_OD.

*Note on nomenclature*: The cinnamaldehyde derivatives 2-methoxycinnamaldehyde (IUPAC name (E)-3-(2-methoxyphenyl)prop-2-enal, also called Cassiastearoptene, because it was isolated for the first time from the essential oil of *C. cassia*) and 2-hydroxycinnamaldehyde (IUPAC name (E)-3-(2-hydroxyphenyl)prop-2-enal) present the substituent (methoxy or hydroxyl group, respectively) in the ortho position.

To acquire ^1^H NMR spectra of samples dissolved in D_2_O, both 1D NOESY-presat and CPMG relaxation-editing sequences with presaturation were tested and 1D NOESY-presat pulse sequence was chosen to obtain more accurate quantitative data (Ciaramelli et al., 2017).

Resonance assignments are annotated in NMR spectra depicted in Figure 1 and Supplementary Figures S2, S3, and in Table 1, that also reports the resonances of unidentified spin systems. Some of them are part of a cinnamaldehyde derivative spin system, and others were assigned to unknown metabolites.

To the best of our knowledge, this is the first time that the metabolic profile of cinnamon buds was characterized by NMR, since only few works on the characterization of the volatile fraction of bud oil are reported in the literature (Jayaprakasha et al., 2002; Kaul et al., 2003). The NMR-driven characterization of cinnamon bark extracts is reported in some papers in methanol or methanol/buffer and our data agree with those previously reported (Lei et al., 2015; Farag et al., 2018). However, the ^1^H-NMR metabolic profile of cinnamon extracts obtained in water is reported here for the first time.

Notably, the aromatic regions of all the spectra showed broad resonances that we can speculate belong to polyphenols. Their composition was further investigated by UPLC-HR-MS, as described in 4.4.

After the manual identification of the compounds reported in Table 1, specific libraries for metabolite semiautomatic identification and quantification were built using the Simple Mixture Analysis (SMA) tool implemented in the MestReNova 14.2.1 software for NMR spectra acquired both in D_2_O and CD_3_OD (Ciaramelli et al., 2018; Ciaramelli et al., 2019; Palmioli et al., 2019; Palmioli et al., 2020; Ciaramelli et al., 2021). The libraries developed with this approach are available as.exp files (Airoldi et al., 2021). An example of SMA output is reported in Supplementary Figure S4.

Tables 2, 3 report the metabolite concentrations obtained from the ^1^H-NMR spectra of samples dissolved in CD_3_OD (all the extraction procedures and all the matrices) and D_2_O (only the water extracts of all the matrices), respectively. In this way, the identity and concentrations of metabolites obtained under different extraction conditions can be directly compared. Moreover, the extraction yields reported in Tables 2, 3 allow the comparison of the extraction efficiency of the different procedures. The best extraction yields were obtained with the hydroalcoholic procedure. Yields obtained for *C. zeylanicum* are lower than those obtained for *C. cassia* samples*,* with extraction yields of buds being slightly higher.

**TABLE 2:** Mean concentrations (M) and corresponding standard deviations (SD) of metabolites contained in cinnamon extracts dissolved in CD_3_OD as determined from ^1^H-NMR spectra. Concentrations of metabolites are expressed in mg/g of extract. M and SD of the extraction yields are also reported. Results are reported as the mean and SD of three independent experiments.

	CZE		CZHE		CZH		CCE		CCHE		CCH		BCE		BCHE		BCH	
	M	SD	M	SD	M	SD	M	SD	M	SD	M	SD	M	SD	M	SD	M	SD
Acetate	8.23	1.25	0.92	0.42	1.57	0.99	2.04	0.22	0.14	0.09	0.68	0.17	2.53	0.99	3.50	0.54	5.92	2.25
Benzoic acid	0.54	0.53	0.70	0.21	0.07	0.10	2.43	0.90	1.39	0.93	0.74	0.81	1.91	1.32	0.65	0.84	0.55	0.41
2,3-Butanediol	2.64	0.31	ND	ND	0.91	0.40	ND	ND	ND	ND	ND	ND	ND	ND	ND	0.21	ND	ND
β-Caryophyllene	27.25	9.01	3.55	2.51	3.98	1.94	14.53	2.49	2.09	1.55	3.11	0.77	28.29	8.70	12.65	3.23	8.02	0.33
Choline	1.05	0.66	1.64	0.46	1.68	0.53	0.46	0.52	1.91	0.17	2.63	0.44	1.42	0.50	2.31	0.80	2.79	0.07
(E)-Cinnamaldehyde	156.65	38.98	9.72	6.59	57.24	21.76	151.31	15.53	33.86	19.54	59.15	9.47	130.93	7.09	49.31	15.29	29.96	8.41
Cinnamaldehyde dimethyl acetal	42.26	10.93	ND	ND	ND	ND	10.36	5.04	3.11	2.23	4.39	1.13	14.26	1.78	ND	ND	3.42	1.70
Cinnamic acid	11.35	5.70	7.12	4.76	4.77	2.31	3.11	2.17	6.47	2.02	4.72	2.43	6.75	5.60	11.24	4.85	10.62	2.27
(Z)-Cinnamaldehyde	2.09	1.48	1.56	2.21	0.81	0.51	0.70	0.44	3.30	2.53	0.54	0.19	0.94	0.21	0.61	0.26	1.51	0.96
Coumarin	0.96	0.68	0.65	0.53	0.63	0.90	ND	ND	ND	ND	ND	ND	ND	ND	ND	ND	ND	ND
Eugenol	14.23	0.70	7.92	0.80	7.65	2.13	ND	ND	ND	ND	ND	ND	ND	ND	ND	ND	ND	ND
Formate	ND	ND	0.93	1.04	0.46	0.45	ND	ND	3.59	1.37	1.06	1.15	1.11	1.31	0.52	0.21	0.59	0.28
Galactose	ND	ND	ND	ND	ND	ND	ND	ND	ND	ND	10.66	3.23	16.62	6.03	ND	ND	22.98	3.61
Glucose	57.97	32.30	27.36	15.77	28.86	8.55	65.78	19.52	68.86	6.93	74.74	20.77	93.96	21.97	72.62	21.11	88.96	1.94
Glycerol	ND	ND	ND	ND	4.44	6.28	19.71	9.14	12.73	0.44	15.84	3.65	9.83	4.79	10.40	5.00	16.99	0.14
2-Hydroxycinnamaldehyde	3.50	2.27	ND	ND	1.37	1.34	1.36	0.46	5.52	3.90	0.67	0.45	0.84	0.30	0.89	0.88	2.04	1.33
Methyl salicylate	19.31	2.57	12.24	2.49	3.46	0.99	28.84	1.85	27.79	1.42	14.98	1.98	24.75	1.82	17.99	1.82	9.75	1.88
2-Methoxycinnamaldehyde	ND	ND	5.43	5.47	2.16	0.68	0.56	0.78	ND	ND	ND	ND	11.94	1.66	5.46	1.96	0.89	0.58
Phenylalanine	91.96	12.19	ND	ND	32.23	8.55	42.99	6.11	ND	ND	14.02	0.27	53.96	4.98	ND	ND	ND	ND
Shikimic acid	21.17	12.12	8.99	4.78	7.92	3.16	6.82	1.76	5.65	0.90	10.39	1.54	21.74	12.33	24.50	12.56	33.63	2.97
Succinate	1.18	0.67	0.94	0.63	1.31	0.59	0.93	0.32	0.71	0.28	1.11	0.38	1.10	0.24	0.87	0.90	1.75	0.22
Sucrose	ND	ND	ND	ND	ND	ND	ND	ND	ND	ND	2.61	2.09	9.24	8.22	6.18	4.40	7.69	1.52
Extraction yield %	5.74	1.09	6.95	1.50	4.43	0.82	11.44	1.36	13.42	3.46	10.01	1.71	16.59	0.61	18.51	2.13	13.07	1.96

ND: not detectable because under the detection limit or in a too crowded spectral area. BC: *Cinnamomum cassia* buds; CC: *Cinnamomum cassia* bark; CZ: *Cinnamomum zeylanicum* bark (CZ). E: alcoholic extraction in ethanol; HE: hydroalcoholic extraction in water (pH 4.5)/ethanol 7:3; H: aqueous extraction.

**TABLE 3:** Mean concentrations (M) and corresponding standard deviations (SD) of metabolites contained in aqueous cinnamon extracts dissolved in D_2_O as determined from ^1^H-NMR spectra. Concentrations of metabolites are expressed in mg/g of extract. M and SD of the extraction yields are also reported. Results are reported as the mean and SD of three independent experiments.

	CZH		CCH		BCH	
	M	SD	M	SD	M	SD
Acetate	1.55	0.60	0.97	0.13	5.27	2.17
Alanine	ND	ND	0.44	0.06	1.76	0.29
Benzoic acid	1.04	0.51	0.40	0.01	1.29	0.22
2,3-Butanediol	0.69	0.10	1.26	0.06	0.90	0.15
(E)-Cinnamaldehyde	16.29	1.14	19.40	10.63	21.06	5.73
Cinnamic acid	3.86	0.91	4.16	0.94	5.99	1.48
Formate	0.55	0.40	0.20	0.09	0.29	0.05
Fructose	31.98	8.80	46.44	6.19	45.82	9.55
GABA (γ-Aminobutyric acid)	6.21	0.91	3.29	0.65	5.21	1.13
Galactose	6.55	0.92	5.22	0.69	4.88	1.18
Glucose	40.88	6.95	69.50	11.31	60.89	10.21
Malic acid	34.69	11.48	ND	ND	ND	ND
*Myo*-inositol	41.22	12.06	39.44	1.98	48.52	11.77
Quinic acid	59.26	14.19	65.72	1.99	73.86	18.77
Shikimic acid	ND	ND	12.96	0.96	27.75	5.81
Succinate	3.41	0.79	2.47	0.35	2.26	0.45
Sucrose	7.09	0.16	ND	ND	5.15	1.72
Extraction yield %	4.43	0.82	10.01	1.71	13.07	1.96

ND: not detectable because under the detection limit or in a too crowded spectral area. BC: *Cinnamomum cassia* buds; CC: *Cinnamomum cassia* bark; CZ: *Cinnamomum zeylanicum* bark (CZ); H: aqueous extraction.

**FIGURE 2:**
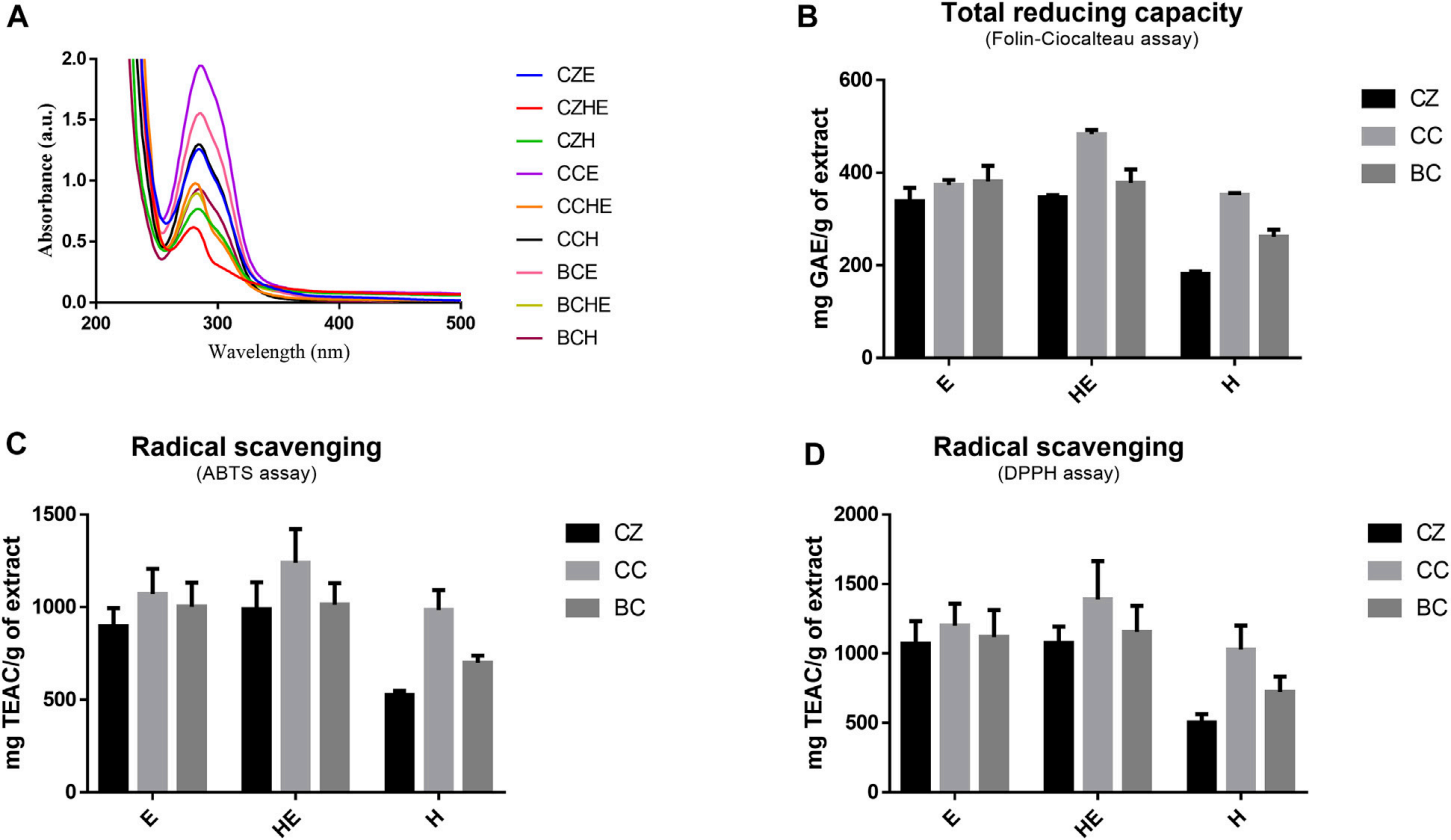
Extract antioxidant activity. UV-Vi’s absorption spectra of cinnamon extracts dissolved at 50 μg/ml in MilliQ water **(A)**. Total reducing capacity (or total polyphenolic content) was assessed by Folin–Ciocalteau’s assay **(B)** and radical scavenging capability was assessed by ABTS and DPPH assays **(C,D)**. Total reducing capability results are expressed as mg of GAE equivalents *per* g of freeze-dried cinnamon extracts and radical scavenging results are expressed as mg of Trolox equivalents (TE) *per* g of freeze-dried cinnamon extracts. All data are reported as mean ± SD of triplicate measurements in three independent experiments. BC: *Cinnamomum cassia* buds; CC: *Cinnamomum cassia* bark; CZ: *Cinnamomum zeylanicum* bark (CZ); E: alcoholic extraction in ethanol; HE: hydroalcoholic extraction in water (pH 4.5)/ethanol 7:3; H: aqueous extraction.

The comparison of NMR spectra (Figure 1) and metabolite concentrations (Tables 2, 3) clearly indicates that (E)-cinnamaldehyde and its derivatives are preferentially extracted in ethanol (Supplementary Figure S5). Eugenol was found only in *C. zeylanicum* samples and not in *C. cassia* barks and buds extracts (Figure 2). This finding agrees with literature data (Rao and Gan, 2014). Notably, *myo-*inositol was detected only after sample extraction in water and extract solubilization in the same solvent (Figure 2). This is a clear consequence of its high polarity and solubility in water.

### 3.2 Cinnamon Extracts’ Antioxidant Capacity

The antioxidant property of cinnamon is one of the main features promoting its use as a potential nutraceutical. Moreover, several compounds with antioxidant ability reduced the oxidative stress induced by Aβ species both *in vitro* and *in vivo*. Treating the cells with different antioxidants can significantly affect proteomic changes due to Aβ-mediated oxidative stress (Cheignon et al., 2018) responsible for cell damage and death.

Thus, we evaluated the total reducing power (or total polyphenolic content) and radical scavenging capacity of our samples by spectrophotometric method assays (Figure 2) (Amigoni et al., 2017; Ciaramelli et al., 2018).

The observation of the UV-visible absorption spectra showed an intense and broad absorption band centered at 286 nm with a shoulder band at 303 nm, typical of cinnamate derivatives, in agreement with their different content in the different extracts (Figure 2A). The evaluation of the total reducing capacity (or total polyphenolic content) and radical scavenging showed a slightly higher antioxidant capacity of extracts obtained by hydroalcoholic extraction (HE) and alcoholic extraction (E), being the best the *C. cassia* hydroalcoholic extract (CCHE), showing an average of 483 mg GAE/g and 1,337 mg TEAC/g (Figures 2B–D).

*C. cassia* buds hydroalcoholic extracts (BCHE) had an average of 378 mg GAE/g and 1,134 mg TEAC/g, whereas the lowest antioxidant capacity was found for the *C. zeylanicum* bark aqueous extract (CZH) with an average of 182 mg GAE/g and 513 mg TEAC/g.

These results highlighted the ability of cinnamon extracts, both from bark and buds, to exert beneficial effects in terms of antioxidant power and radical scavenging activity.

### 3.3 Cinnamon Extracts’ Ability to Counteract Aβ1-42 Peptide Aggregation and Neurotoxicity

**FIGURE 3:**
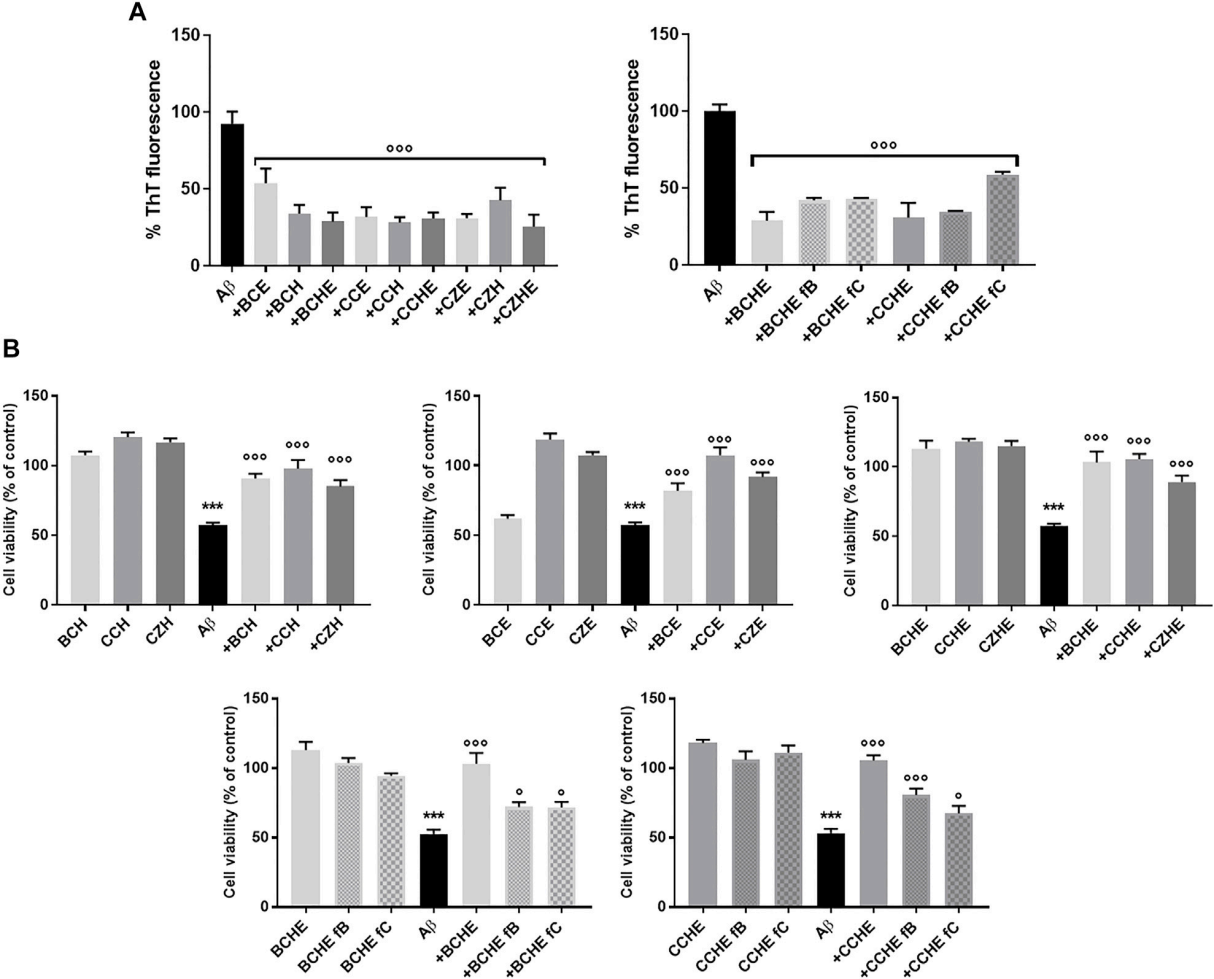
Effects of cinnamon extracts and fractions on Aβ1-42 aggregation and oligomers toxicity on the human neuroblastoma SH-SY5Y cell line. **(A)** The effect of incubation (24 h at 37°C) of BC, CC, and CZ extracts (2.5 μg/ml) and BCHE or CCHE fractions B and C (2.5 μg/ml) on Aβ1-42 (2.5 μM) aggregation was determined by ThT fluorescence assay. Values are the mean ± standard deviation of three replicates after subtracting their relative control solutions (extracts alone). ***p < 0.001 vs. Aβ1-42 alone. Dunnett’s multiple comparisons test followed one-way ANOVA. **(B)** Cells were treated with Aβ1-42 peptide (10 μM) and incubated with or without 10 μg/ml of BC, CC, and CD extracts or BCHE and CCHE fractions B and C for 24 h, and the toxicity was evaluated by MTT assay. Values are the mean ± standard deviation of three replicates. One-way ANOVA followed by Dunnett’s multiple comparison test: ***p < 0.001 vs. vehicle, *p < 0.05 and ***p < 0.001 vs. Aβ1-42 alone. BC: *Cinnamomum cassia* buds; CC: *Cinnamomum cassia* bark; CZ: *Cinnamomum zeylanicum* bark (CZ); fB: fraction B; fC: fraction C; E: alcoholic extraction in ethanol; HE: hydroalcoholic extraction in water (pH 4.5)/ethanol 7:3; H: aqueous extraction.

Cinnamon extracts were characterized for their ability to inhibit the aggregation of Aβ1-42 peptides by Thioflavin T (ThT) assay (Hawe et al., 2008). ThT is a benzothiazole dye that gives enhanced fluorescence upon binding to amyloid aggregates *in vitro* and *ex vivo* and is usually employed to monitor the aggregation of Aβ peptides and quantify the formation of amyloid aggregates in the absence or presence of anti-amyloidogenic compounds. Aβ1-42 peptide (2.5 μM) was incubated for 24 h at 37 °C with 2.5 μg/ml of extracts BC, CC, and CZ. The treatment with all the extracts reduced the peptide aggregation (reduction from 46.5% to 75%) (Figure 3A).

To evaluate the ability of cinnamon extracts to reduce the Aβ1-42 cytotoxicity, the human neuroblastoma SH-SY5Y cell line was treated for 24 h with Aβ1-42 peptide and BC, CC, and CZ extracts. The reduction of cell viability was 43% (*p* < 0.001 vs. vehicle) after cell incubation with 10 μM of Aβ1-42 peptide. Cinnamon extracts counteracted this effect when added at a concentration of 10 μg/ml, determining a significant increase of cell survival (from 27.7% to 40.5% for H extraction; from 24.5% to 50.1% for E extraction; from 31.7% to 48.2% for HE extraction, *p* < 0.001 vs. Aβ1−42 alone) (Figure 3B).

**FIGURE 4:**
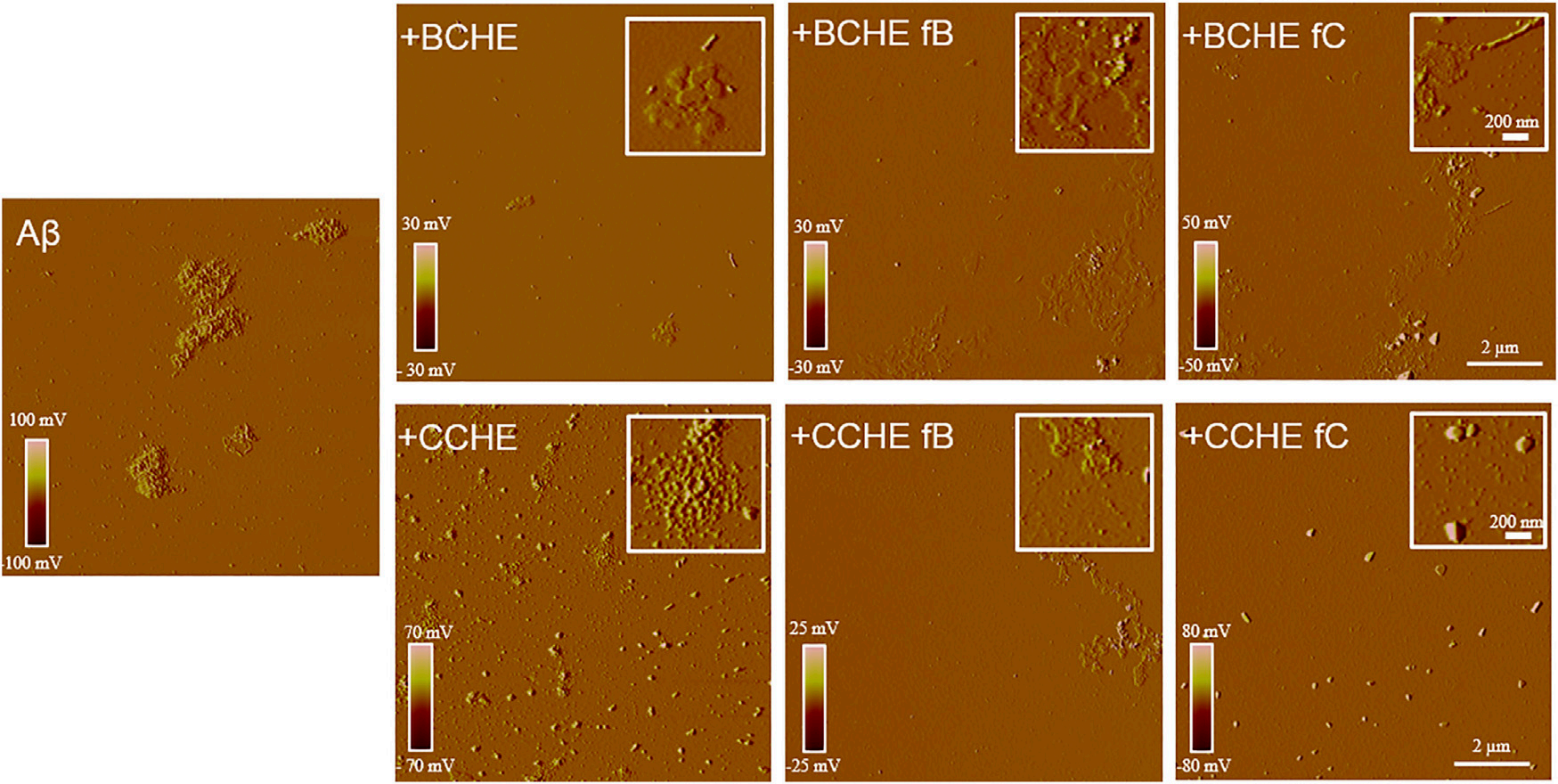
Effects of cinnamon extracts and fractions on Aβ1-42 aggregates’ morphology. AFM images were acquired after 24 h incubation at 37°C of Aβ 1–42 peptide (2.5 μM) with or without cinnamon extracts BCHE and CCHE and related fractions B and C (2.5 µg/ml). BC: *Cinnamomum cassia* buds; CC: *Cinnamomum cassia* bark; E: alcoholic extraction in ethanol; HE: hydroalcoholic extraction in water (pH 4.5)/ethanol 7:3; fB: fraction B; fC: fraction C.

BCHE and CCHE are among the most potent in inhibiting peptide aggregation and preventing its toxicity (Figure 3) and their activity was further evaluated by Atomic Force Microscopy (AFM) morphological analysis of Aβ aggregates. AFM images (Figure 4) showed that the cinnamon extracts BCHE and CCHE affect the aggregation of Aβ1−42 peptide

In particular, all the extracts coincubated with the peptide lead to the formation of abundant amorphous material. In the presence of BCHE extracts, a very small part of the peptide evolves towards short protofibrils, while when the Aβ1-42 peptide was coincubated with CCHE extracts, it evolves towards roundish clustered structures of large dimensions not able to bind the ThT, indicating the absence of structures in β-sheets.

### 3.4 *C. Cassia* Hydroalcoholic Extract and Buds Hydroalcoholic Extracts Fractionation and UPLC-HRMS Characterization of Their Polyphenolic Content

To investigate the correlation between the anti-amyloidogenic activity of CCHE and BCHE and their metabolite content deeply, we fractioned these extracts by preparative reverse-phase (RP) C18 flash chromatography.

**FIGURE 5:**
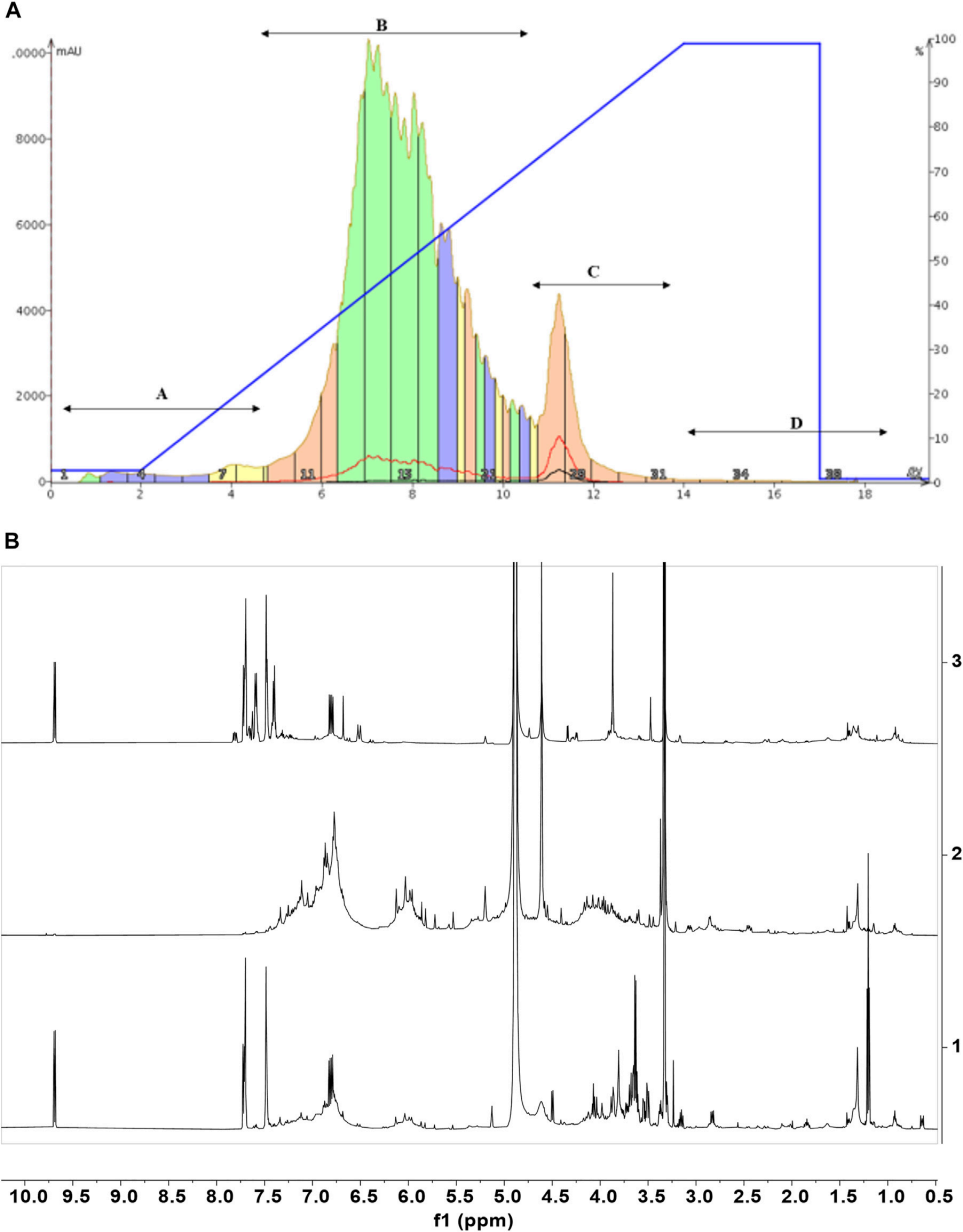
Cinnamon extracts’ fractionation. Chromatographic profile **(A)** of the separation of CCHE extract obtained by reverse phase C18 chromatography (linear elution gradient from 2% to 100% MeOH in 15 CV). **(B)** The ^1^H-NMR spectra of the chromatographic fractions B (2) and C (3) compared to the ^1^H-NMR profile of the total extract (1). ^1^H-NMR spectra were recorded on 5 mg/ml samples dissolved in CD_3_OD, 25°C, at 600 MHz.

The chromatography and analysis of fractions obtained from CCHE are reported, as an example, in Figure 5. The chromatographic profile is depicted in Figure 5A. Fractions B and C were collected and concentrated on the bases of absorbance. Then they were analyzed by NMR spectroscopy (Figure 5B).

**FIGURE 6:**
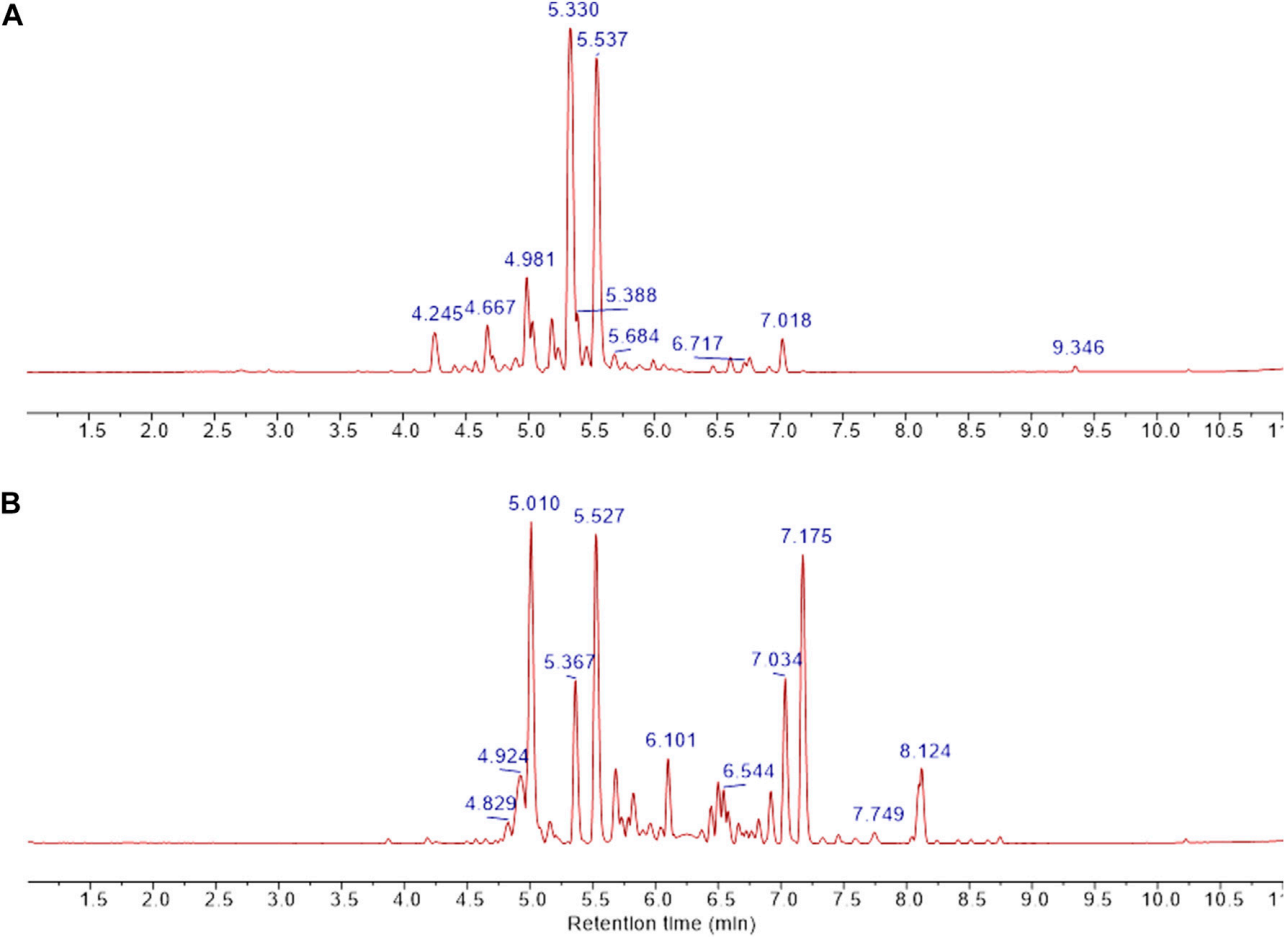
UPLC separation of fractions B was obtained from CCHE and BCHE samples. Base peak extracted chromatograms of fraction B were obtained from CCHE **(A)** and BCHE **(B)**. UPLC separation was performed on Waters Acquity Premier HSS T3 column (100 × 2.1 mm I.D., 1.8 μm) applying a linear elution gradient from 5% to 50% B in 10 min at a flow rate of 0.4 ml/min (A: H_2_O, 0.1% formic acid, **(B)** MeCN, 0.1% formic acid). Retention times were reported on significant peaks.

The NMR characterization was enough to identify metabolites contained in fraction C. This analysis unveiled cinnamaldehyde and its derivatives as the most abundant molecules. In the case of fraction C from BCHE extract, a very small amount of polyphenols was also detected (data not shown). The NMR characterization of fractions B from BCHE and CCHE indicated the presence of resonance characteristic of polyphenols, also in glycosylated forms. A comparison among the NMR spectra of the two fractions B showed remarkable differences (Supplementary Figure S7), suggesting the presence of slightly different molecular species, but their identification was not possible due to the significant resonance overlapping. Thus, they were fully analyzed by UPLC-HR-MS (Figure 6 and Tables 4, 5).

**TABLE 4:** UPLC/HR-MS data for the major extract components identified in fraction B was obtained from the CCHE extract.

(min)	RT	Name	Molecular formula	Monoisotopic mass	Experimental HRMS [M-H]^-^	Abs. error (ppm)
1	4.245	B-type procy dimer	C30H26O12	578.1424	577.1354	0.5
2	4.27	A-type procy tetramer	C60H48O24	1,152.2540	1,151.245	1.11
3	4.412	Unkn (phenolic glycoside)	C19H28O12	448.1581	493.1564^a^	0.31
4	4.486	B-type procy dimer	C30H26O12	578.1424	577.1348	0.56
5	4.578	Unkn (O-glycosyl compounds)	C18H26O11	418.1475	417.1404	0.42
6	4.667	Epicatechin	C15H14O6	290.0790	289.0716	0.47
7	4.714	B-type procy trimer	C45H38O18	866.2058	865.1981	0.55
8	4.808	B-type procy trimer	C45H38O18	866.2058	865.1969	1.89
9	4.892	A-type procy pentamer	C75H60O30	1,440.3170	1,440.3209^b^, 719.1532^c^	2.79
10	4.981	A-type procy tetramer	C60H48O24	1,152.2540	575.1226^c^, 1,151.246	0.26
11	5.031	B-type procy dimer	C30H26O12	578.1424	577.1357	1.02
12	5.183	Benzyl beta-primeveroside	C18H26O10	402.1526	447.1520^a^, 401.1451	0.45
13	5.236	A-type procy pentamer	C75H60O30	1,440.3170	1,440.3233^b^, 719.1524^c^	1.68
14	5.33	A-type procy trimer	C45H36O18	864.1902	863.1837	0.89
15	5.388	catechin	C15H14O6	290.0790	289.0718	0.05
16	5.456	A-type procy tetramer	C60H48O24	1,152.254	575.1222^c^, 1,151.245	1.11
17	5.537	A-type procy trimer	C45H36O18	864.1902	863.1846	2.02
18	5.684	A-type procy tetramer	C60H48O24	1,152.2540	575.1234^c^, 1,151.2469	0.95
19	5.768	B-type procy dimer	C30H26O12	578.1424	577.1357	1.02
20	5.878	A-type procy trimer	C45H36O18	864.1902	863.1832	0.39
21	5.988	Phenylethyl primeveroside	C19H28O10	416.1682	461.1678^a^, 415.1614	1.12
22	6.075	A-type procy trimer	C45H36O18	864.1902	863.1827	0.17
23	6.462	B-type procy dimer	C30H26O12	578.1424	577.1351	0.14
24	6.580	Ptelatoside B	C20H28O10	428.1682	473.1666^a^	0.37
25	6.607	A-type procyanidin	C45H34O18	862.1745	861.1676	0.46
26	6.717	A-type procy dimer	C30H24O12	576.1268	575.1193	0.31
27	6.759	Unkn (phenolic glycoside)	C20H28O10	428.1682	473.1666^a^, 427.1601	0.37
28	6.916	Phenethyl rutinoside	C20H30O10	430.1839	475.1819^a^, 429.176	1.45
29	7.018	Poncirin chalcone	C28H34O14	594.1949	593.187	1.06
30	9.346	Unkn	C22H30O8	422.1941	421.1865	0.78

^a^Deconvoluted;

b [M-2H]^2-^;

c [M + FA-H]^-^.

**TABLE 5:** UPLC/HR-MS data for the major extract components identified in fraction B was obtained from the BCHE extract.

#	RT (min)	Name	Molecular formula	Monoisotopic mass	Experimental HRMS [M-H]^-^	Abs. error (ppm)
1	4.829	B-type Procy trimer	C45H38O18	866.2058	865.1968	1.79
2	4.924	B-type Procy dimer	C30H26O12	578.1424	577.1345	1.09
3	5.01	B-type Procy dimer	C30H26O12	578.1424	577.1352	0.09
4	5.162	B-type Procy tetramer	C60H50O24	1,154.2690	1,153.2598	1.87
5	5.367	Catechin	C15H14O6	290.0790	289.0716	0.68
6	5.527	B-type Procy trimer	C45H38O18	866.2058	865.1997	1.35
7	5.684	B-type Procy tetramer	C60H50O24	1,154.2690	1,153.2640	1.83
8	5.731	B-type Procy pentamer	C75H62O30	1,442.3330	1,442.3341^a^, 720.1598^b^	0.69
9	5.784	B-type Procy trimer	C45H38O18	866.2058	865.1985	0.01
10	5.825	B-type Procy pentamer	C75H62O30	1,442.3330	1,442.3373^a^, 720.1614^b^	2.89
11	5.904	B-type Procy hexamer	C90H75O36	1731.4040	1730.3992^a^, 864.1923^b^	1.54
12	5.962	B-type Procy hexamer	C90H75O36	1731.4040	1730.3994^a^, 864.1924^b^	1.68
13	6.043	B-type Procy heptamer	C105H86O42	2018.4590	2018.4561^a^, 1,008.2208	1.9
14	6.101	Phenylethyl primeveroside	C19H28O10	416.1682	415.1612	0.54
15	6.368	B-type Procy trimer	C45H38O18	866.2058	865.1979	0.79
16	6.441	B-type Procy dimer	C30H26O12	578.1424	577.1352	0.07
17	6.499	Isoquercitrin	C21H20O12	464.0955	463.0887	0.98
18	6.544	Unkn	C25H38O11	514.2414	513.2333	1.55
19	6.58	Ptelatoside B	C20H28O10	428.1682	473.1667^C^	0.63
20	6.664	Quercetin 3-xylosyl-(1->2)-alpha-L-arabinofuranoside	C25H26O15	566.1272	565.1201	0.29
21	6.764	Unkn (Flavonol glycoside)	C39H34O13	710.1999	709.1921	0.74
22	6.822	Rosavin	C20H28O10	428.1682	427.1614	1.02
23	6.921	Avicularin	C20H18O11	434.0849	433.0781	0.99
24	6.926	Astragalin	C21H20O11	448.1006	447.0932	0.19
25	7.034	Quercetin-3-O-deoxyhexosyl(1–2)pentoside	C26H28O15	580.1428	579.1353	0.5
26	7.175	Quercitrin	C21H20O11	448.1006	447.0929	0.87
27	7.333	Juglalin	C20H18O10	418.0900	417.082	1.72
28	7.453	Unkn (phenolic glycoside)	C20H28O10	428.1682	427.1605	1.19
29	7.592	Unkn (Kaempferol-O-glycoside)	C26H28O14	564.1479	563.1399	1.31
30	7.749	Unkn	C39H34O13	710.1999	709.1918	1.17
31	7.854	Secoisolariciresinol	C20H26O6	362.1729	407.1704^C^	1.87
32	8.041	Unkn (Flavanone glycoside)	C24H22O7	422.1366	421.1289	0.88
33	8.093	Cinnamic acid	C9H8O2	148.0524	147.0448	2.28
34	8.119	Unkn	C21H28O10	440.1682	439.1617	1.55

a Deconvoluted.

b [M-2H]^2-^.

c [M + FA-H]^-^.

UPLC separation coupled with high resolution (HR) data-dependent tandem mass scan acquisition (DDA) allows detailed profiling of polyphenols-enriched fractions obtained from CC bark and bud. The base-peak chromatograms (BPCs) are shown in Figures 6A, B and spectrometric data are reported in Tables 4, 5. The main components identification was performed taking into account the experimental accurate mass, isotopic pattern, and fragmentation profile in comparison to literature (Lin et al., 2014; Wang et al., 2020) and public databases.

Overall, these data allowed the identification of flavan-3-ol and procyanidins oligomers, phenolic and flavonoids glycosides. Procyanidin oligomers, ranging from dimers to heptamers, represented the main components in the samples. CCHE fraction B was rich in A-type and B-type procyanidins, whereas only B-type procyanidins were found in BCHE fraction B. Moreover, several glycosyl flavonols, mainly quercetin and kaempferol derivatives, were only found in BCHE fraction B, including quercitrin, isoquercitrin, avicularin, astragalin, and juglalin.

### 3.5 Fractions of *C. Cassia* Hydroalcoholic Extract and Buds Hydroalcoholic Extracts Enriched in Procyanidins and Cinnamaldehyde and Derivatives Inhibit Aβ1-42 Peptide Aggregation and Reduce Aβ1-42-Induced Neurotoxicity

The biological activity of fractions B and C was then tested. Their ability to affect Aβ protein aggregation and Aβ-induced neurotoxicity was tested with thioflavin T (ThT), AFM, and MTT assays, as described in Section 3.3. Aβ1-42 peptide (2.5 μM) was incubated for 24 h at 37°C with 2.5 μg/ml of extracts BCHE and CCHE, with their fractions B and C (Figure 3A and Supplementary Figure S8). The curves obtained (Supplementary Figure S8) showed a strong reduction in the elongation process due to the interference in the nucleation, probably caused by the dispersion of monomers or the inhibition of the fibril’s formation. The treatment with the fractions compared to the associated extracts confirmed the reduction of peptide aggregation. AFM analysis (Figure 4) confirmed their ability to prevent the on-pathway amyloidogenic aggregation of the peptide.

Then, fractions B and C of BCHE and CCHE extracts were tested as inhibitors of Aβ1-42 cytotoxicity. Cells were incubated for 24 h with 10 μM of Aβ1-42 peptide. The reduction of cell viability was 47% (p < 0.001 vs. vehicle), and unlike the relative extracts, the fractions increase cell viability to a lesser and variable extent, depending on the predominant component in the fraction (BC: 19.3% and 19.2%, CC: 27.6% and 14.4% respectively for fraction B and C, *p* < 0.05 and *p*< 0.001 vs. Aβ1-42 alone) (Figure 3B). This indicates that the observed anti-amyloidogenic activity is due to both flavonoids contained in fraction B and cinnamaldehydes contained in fraction C, but we can speculate that, in the total extracts, they have additive or synergistic effects.

### 3.6 Flavonoids and Cinnamaldehydes in Cinnamon Extracts Interact With Aβ1–42 Oligomers

**FIGURE 7:**
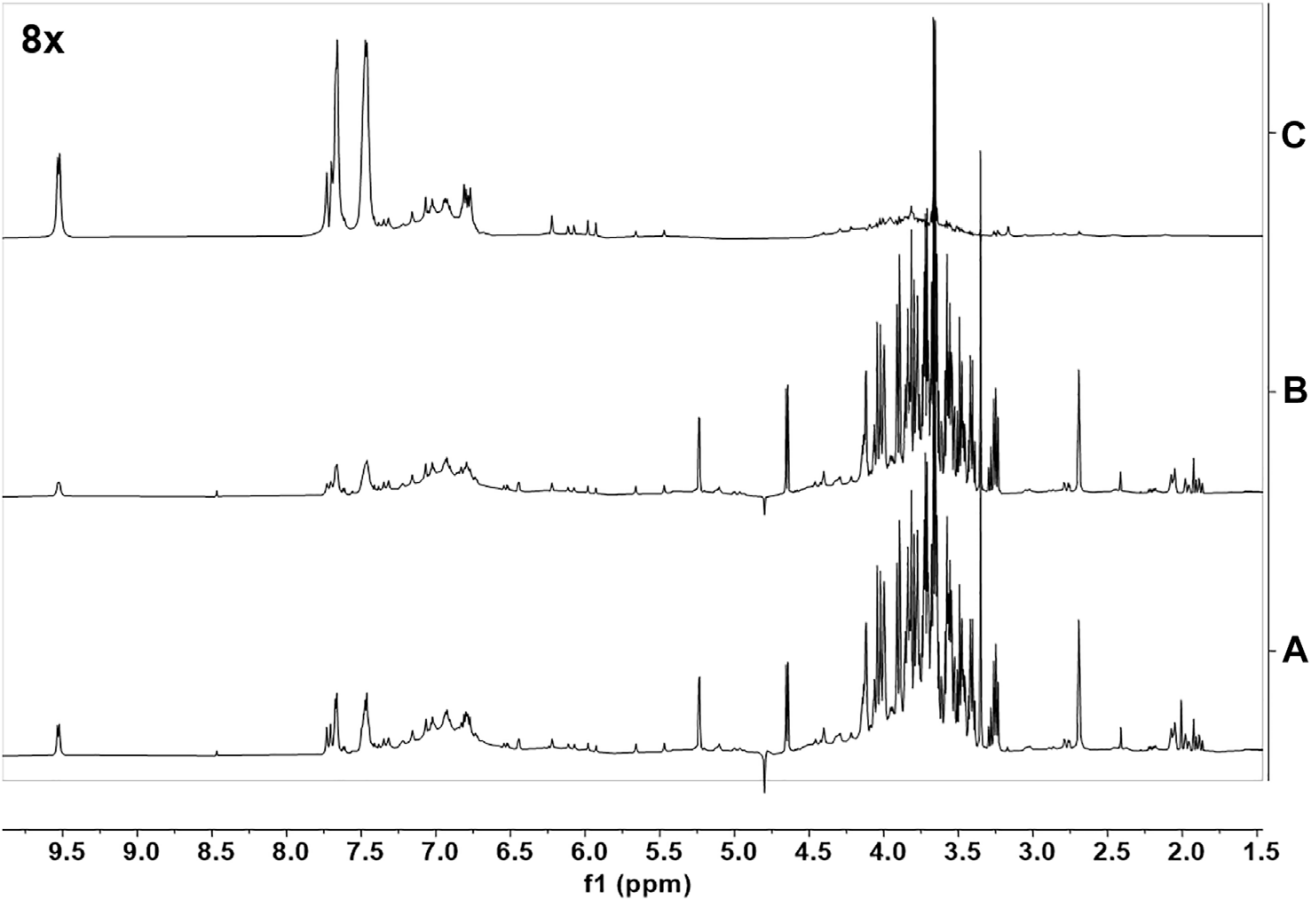
NMR binding studies with Aβ1-42 oligomers. **(A)** The ^1^H-NMR spectrum of a solution containing CCHE extract (15 mg/ml); **(B)** ^1^H-NMR spectrum of a solution containing CCHE extract (15 mg/ml) and Aβ1-42 protein (120 μM); **(C)** STD NMR spectrum of the same sample of **(B)**. Samples were dissolved in deuterated phosphate buffer, pH 7.4. STD spectra were acquired with 1,024 scans and 2 s saturation time at 600 MHz, 25°C.

The identification of the cinnamon extract components that interact directly with Aβ oligomers was based on STD NMR experiments (Mayer and Meyer, 1999), which is a very robust, sensitive, and reliable approach for the screening of pure compounds (Airoldi et al., 2011b; Guzzi et al., 2017), small compound libraries (Airoldi et al., 2011a) or complex mixtures (Airoldi et al., 2011b; Airoldi et al., 2013; Sironi et al., 2014; Ciaramelli et al., 2018; Palmioli et al., 2019; Ciaramelli et al., 2021) aimed at the identification of Aβ oligomers’ ligands. STD spectra were acquired on a mixture containing Aβ oligomers and the CCHE extract (Figures 7A, B, C). To obtain Aβ oligomers, the Aβ1–42 peptide was dissolved in an aqueous phosphate buffer according to the procedure previously described (Airoldi et al., 2011b). To perform STD experiments we selectively saturated some aliphatic protons of Aβ oligomers by irradiating the sample at −1.00 ppm (on-resonance frequency). If Aβ ligand(s) are in solution, a magnetization transfer occurs from the receptor to the ligand(s) protons, and ligand(s) NMR signals can be detected in STD spectra (Figure 7C). A blank experiment (data not shown) was acquired under the same experimental conditions on a sample containing only the crude CCHE extract, to confirm that signals in the STD spectra can be associated to real ligand binding events. Signals belonging to nonbinding compounds did not appear in the STD spectrum. The assignment of resonances appearing in the STD spectra (Figure 7C) to cinnamaldehydes, and other flavonoids indicates their direct interaction with Aβ1-42 oligomers.

STD experiments were then performed on CCHE fraction B (Supplementary Figure S9). Unfortunately, due to signals’ overlapping, also in this case the assignment of compound resonances was not possible, and thus the unambiguous identification of Aβ1-42 oligomers’ ligands contained in fraction B. However, this experiment confirmed that several flavonoids contained bind peptide oligomers. Some of these compounds, such as quercetin and derivatives, have already been reported as Aβ1-42 binders and inhibitors (Guzzi et al., 2017). Moreover, due to the great abundance of procyanidins in this sample, their structural correlation with catechins, already described as very good binders of Aβ peptides and oligomers (Sironi et al., 2014; Airoldi et al., 2018b; Martinez Pomier et al., 2020; Ciaramelli et al., 2021; Ahmed et al., 2022) and the compatibility of their resonances with those appearing in STD spectra of fractions B (Supplementary Figure S9), we can speculate that also these species are good ligands and, according to biological data (see Section 3.4), inhibitors of Aβ oligomers. Our conclusion is supported by the recent literature (Li et al., 2018).

### 3.7 Anti-Aβ Activity of Cinnamon Extracts is Independent of the Autophagy Induction

**FIGURE 8:**
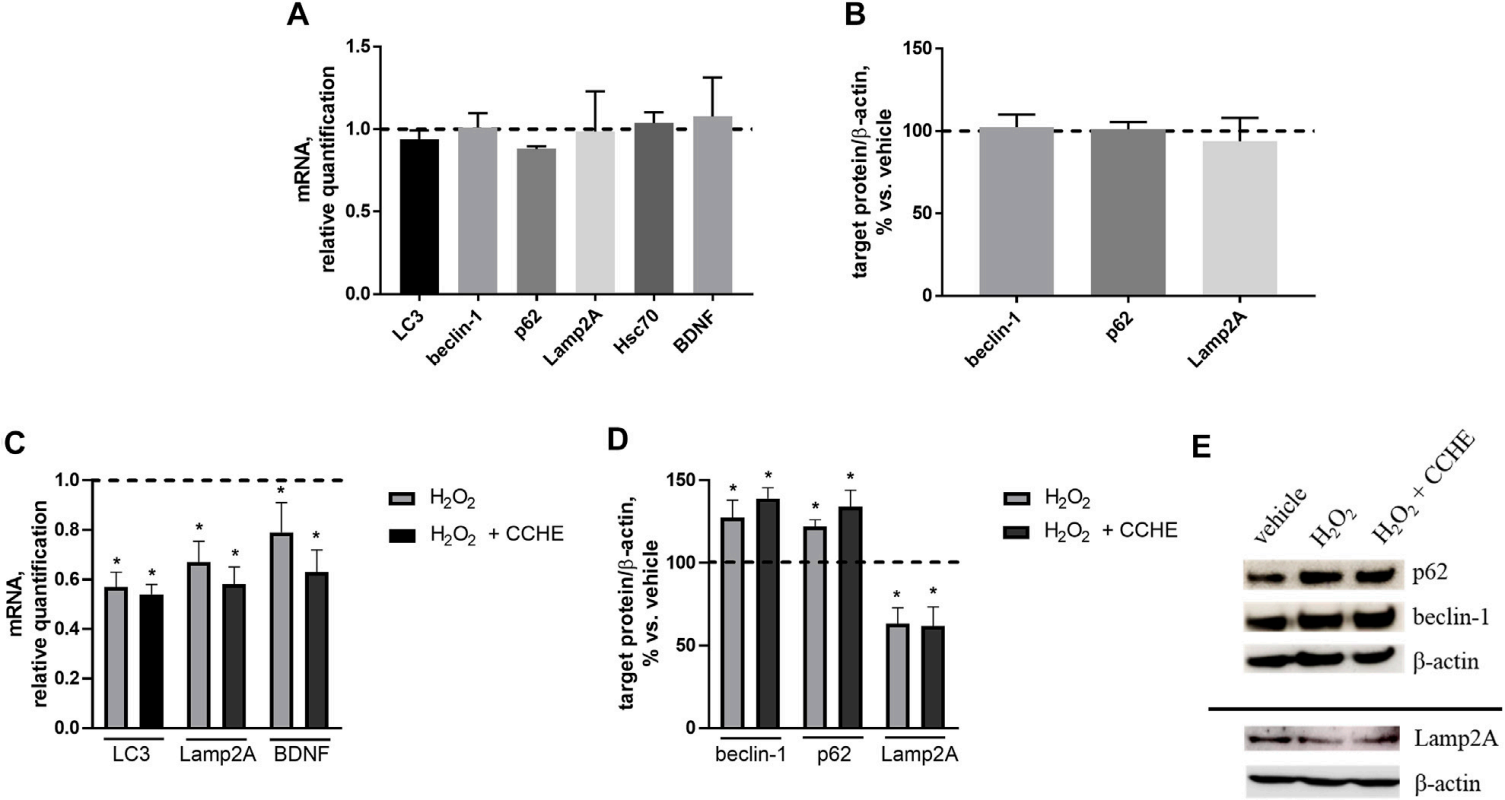
**(A,B)**. Effect of cinnamon extract CCHE (10 µg/ml, 24 h) on autophagy markers in human SH-SY5Y cells under standard culture conditions. **(A)** Relative mRNA levels of macroautophagy (LC3, beclin-1, and p62) and CMA (Lamp2A and Hsc70) markers and BDNF. **(B)** Protein expression of macroautophagy (beclin-1 and p62) and CMA (Lamp2A) markers. **(C–E)**. Effect of cinnamon extract CCHE on autophagy markers in human SH-SY5Y cells exposed to hydrogen peroxide (H_2_O_2_). Cells were pretreated with CCHE (10 µg/ml) for 1 h and then cotreated with H_2_O_2_ (50 μM, 24 h. **(C)** Relative mRNA levels of macroautophagy (LC3) and CMA (Lamp2A) markers and BDNF. **(D)** Protein expression of macroautophagy (beclin-1 and p62) and CMA (Lamp2A) markers and **(E)** representative Western blot image showing immunoreactivity for the target proteins and the corresponding β-actin, used as internal standard. *N* = 3, *p* < 0.05 vs. vehicle-treated cells.

We wondered if autophagy potentiation could mediate cinnamon extracts’ protective role against Aβ aggregation and toxicity. To investigate this point, the expression of the key proteins of the main autophagic pathways, macroautophagy and chaperone-mediated autophagy (CMA), involved in Aβ clearance was assessed. Human neuroblastoma SH-SY5Y cells were exposed for 24 h to 10 µg/ml cinnamon extract (CCHE), and mRNA levels of three macroautophagy (LC3, beclin-1, and p62) and two CMA (Lamp2A and Hsc70) effectors were measured by real-time PCR. Exposure to CCHE extract does not significantly alter the gene expression of any of the autophagy-related targets (Figure 8A). No effect of CCHE extract was also observed on gene expression of the neurotrophic factor BDNF, used as a sensor of neuroprotective effect (Figure 8A). Further reinforcing these results, no effect of CCHE extract was also observed on protein expression of both macroautophagy (beclin-1 and p62) and CMA (Lamp2A) markers (Figure 8B) evaluated by Western blot.

Then, the effect of CCHE extract on autophagy markers was also verified in the same cell line exposed to a well-known pro-oxidant stimulus represented by hydrogen peroxide (H_2_O_2_, 50 µM for 24 h). In this experimental setting, cells were pretreated for 1 h with 10 µg/ml CCHE extract and then cotreated for 24 h with 50 µM H_2_O_2_. As expected, H_2_O_2_ exposure down-regulates the gene expression of macroautophagy (LC3) and CMA (Lamp2A) markers, as well as BDNF (Figure 8C). Also in this condition, CCHE extract was unable to counteract the effect of H_2_O_2_ on these parameters (Figure 8C). Accordingly, the exposure to CCHE extract did not modify the alterations of macroautophagy (beclin-1 and p62) and CMA (Lamp2A) protein levels induced by H_2_O_2_ (Figure 8D, E).

Collectively, these results indicate that the protective role of cinnamon extracts demonstrated in this study at the used concentration (10 µg/ml) and time exposure (24 h) cannot be ascribed to potentiation of autophagic pathways in SH-SY5Y cells, both under standard culture conditions and in the presence of a pro-oxidant condition.

## 4 Discussion

For the first time, we report the ^1^H-NMR characterization of cinnamon bud metabolic profile. We compared the bud profile with that of cinnamon bark of the same species, *C. cassia*, and that of *C. zeylanicum.* Moreover, we characterized their polyphenols’ content in detail by UPLC-HR-MS, highlighting B-type procyanidins as the main components of the flavonoid family, at variance with bark, richer in A-type procyanidins. These data provide a deeper investigation of the natural compounds present in cinnamon buds, previously characterized only in their volatile components by GC-MS analysis.

Moreover, by combining biophysical, biochemical, and NMR spectroscopy experiments, we identified flavanols, particularly procyanidins and cinnamaldehydes, as ligands and inhibitors of Aβ1-42 aggregation and cytotoxicity.

This direct effect against Aβ peptide adds to the previously reported (Peterson et al., 2009) ability of procyanidin and cinnamaldehyde to inhibit tau protein aggregation, another important hallmark of AD (Medeiros et al., 2011).

Notably, the lack of effect of cinnamon extract on autophagy suggested that, in our experimental model and conditions, cinnamon anti-AD activity is mainly due to the ability of its molecular components (flavanols and cinnamaldeydes in particular) to hinder the aggregation of amyloidogenic proteins, thus preventing their cytotoxic effects.

Moreover, thanks to their significant antioxidant activity, cinnamon extracts can also reduce the Aβ-mediated oxidative stress that decreases cell viability in several cell lines, including SH-SY5Y (Cheignon et al., 2018).

Collectively, our results suggest that cinnamon is a rich source of natural bioactive compounds able to exert a multitarget activity against AD

Both bark and the less-studied bud extracts could be useful ingredients for the preparation of nutraceuticals and functional foods for the prevention of AD through the regular intake of natural compounds that interfere with Aβ and tau aggregation and related neurotoxicity.

At the same time, in addition to catechins, procyanidins, as already reported in recent studies (Mizuno et al., 2019; Zhao et al., 2019; Ruan et al., 2021), can be further investigated as potential molecular templates for the development of new anti-amyloidogenic drugs.

## Data Availability

The datasets presented in this study can be found in online repositories. The names of the repository/repositories and accession number(s) can be found below: https://board.unimib.it/datasets/v3kj6zn9tf/1, Mendeley Data, V1, doi: 10.17632/v3kj6zn9tf.1.
