# Supplementary material for: NMR-Driven Identification of Cinnamon Bud and Bark Components With Anti-Aβ Activity
Source: Front Chem. 2022 Jun 8;10:896253. doi: 10.3389/fchem.2022.896253 (PMC9214034; doi:10.3389/fchem.2022.896253)
Supplement: Supplementary file 1 [file DataSheet1.docx]

Supplementary Material


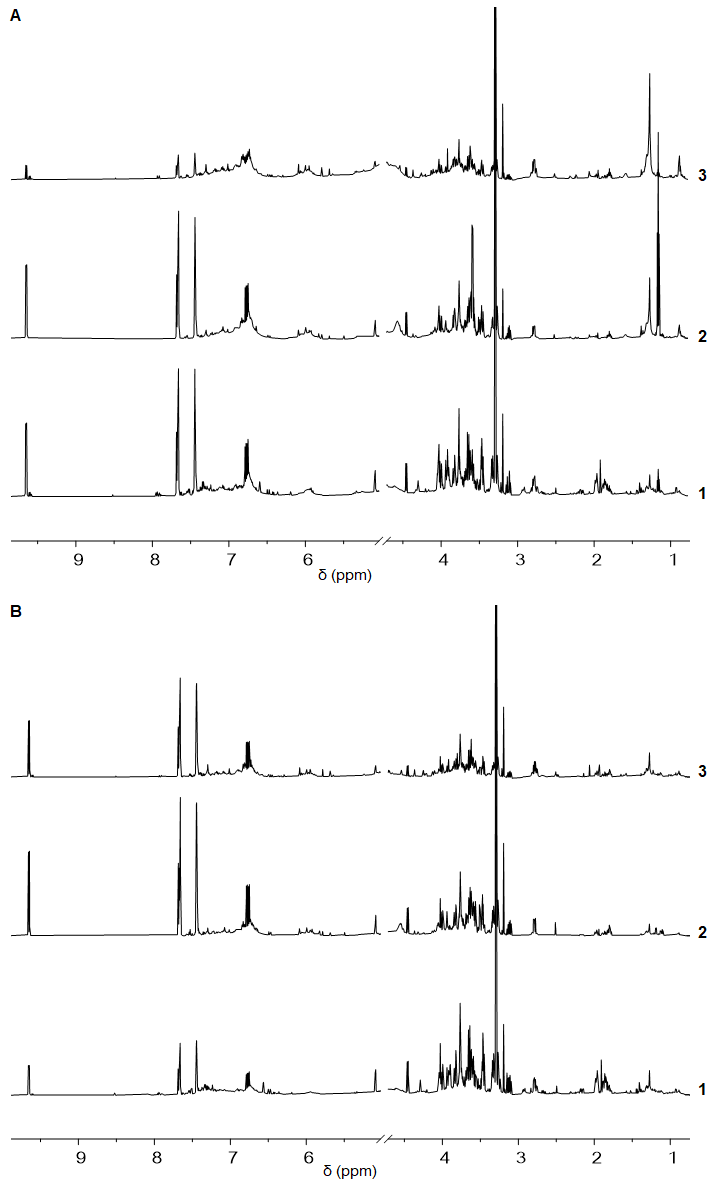


**Supplementary Figure S1.** ^1^H-NMR spectra of extracts of cinnamon buds (**1**) and bark (**2**, *C. cassia;* **3**, *C* *zeylanicum*) obtained with different extraction solvents: hydroalcoholic with water/ethanol (**A**) and acid water (**B**). ^1^H-NMR spectra were recorded on 15 mg/mL samples dissolved in CD_3_OD with 1 mM DSS, with *noesygppr1d* acquisition pulse sequence, at 600 MHz, 25 °C.


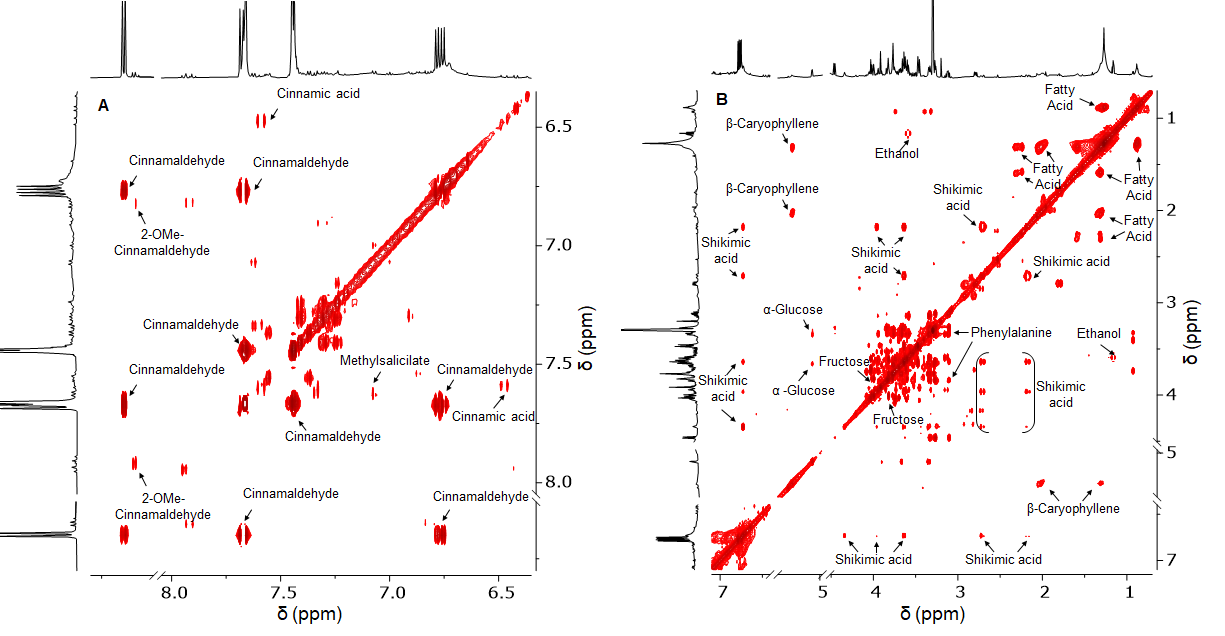


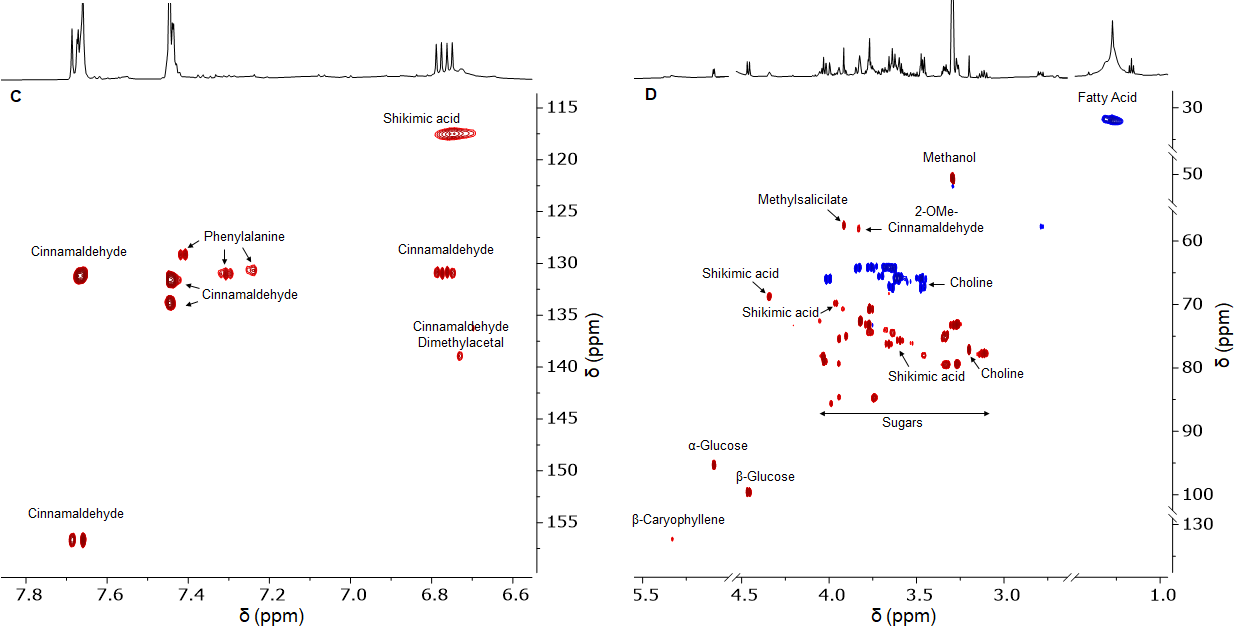


**Supplementary Figure S2**. Expansions of ^1^H,^1^H-TOCSY (**A** and **B**) and ^1^H,^13^C-HSQC (**C** and **D**) spectra of BCE extract in CD_3_OD. ^1^H-NMR spectrum is reported along the axes in the ^1^H, ^1^H-TOCSY and along f2 in the ^1^H,^13^C-HSQC. The resonances of some of the most important metabolites are reported on the 2D spectra.


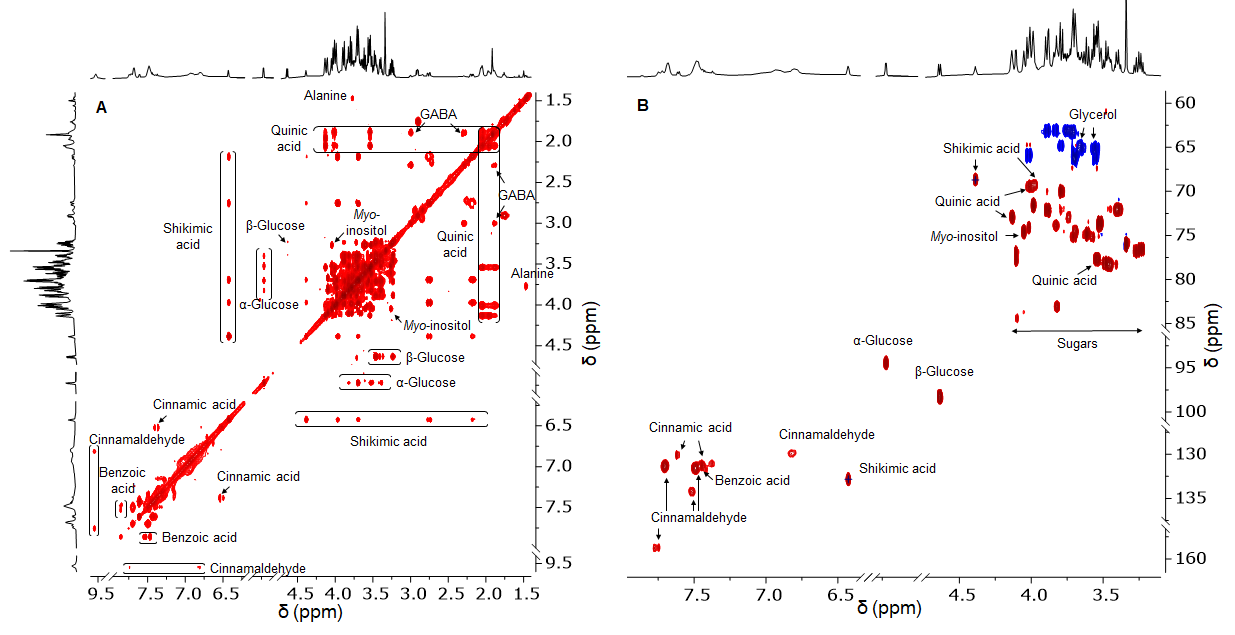


**Supplementary Figure S3**. Expansions of ^1^H,^1^H-TOCSY (**A**) and ^1^H,^13^C-HSQC (**B**) spectra of BCH extract in D_2_O. ^1^H-NMR spectrum is reported along the axes in the ^1^H,^1^H-TOCSY and along f2 in the ^1^H,^13^C-HSQC. The resonances of some of the most important metabolites are reported on the 2D spectra.


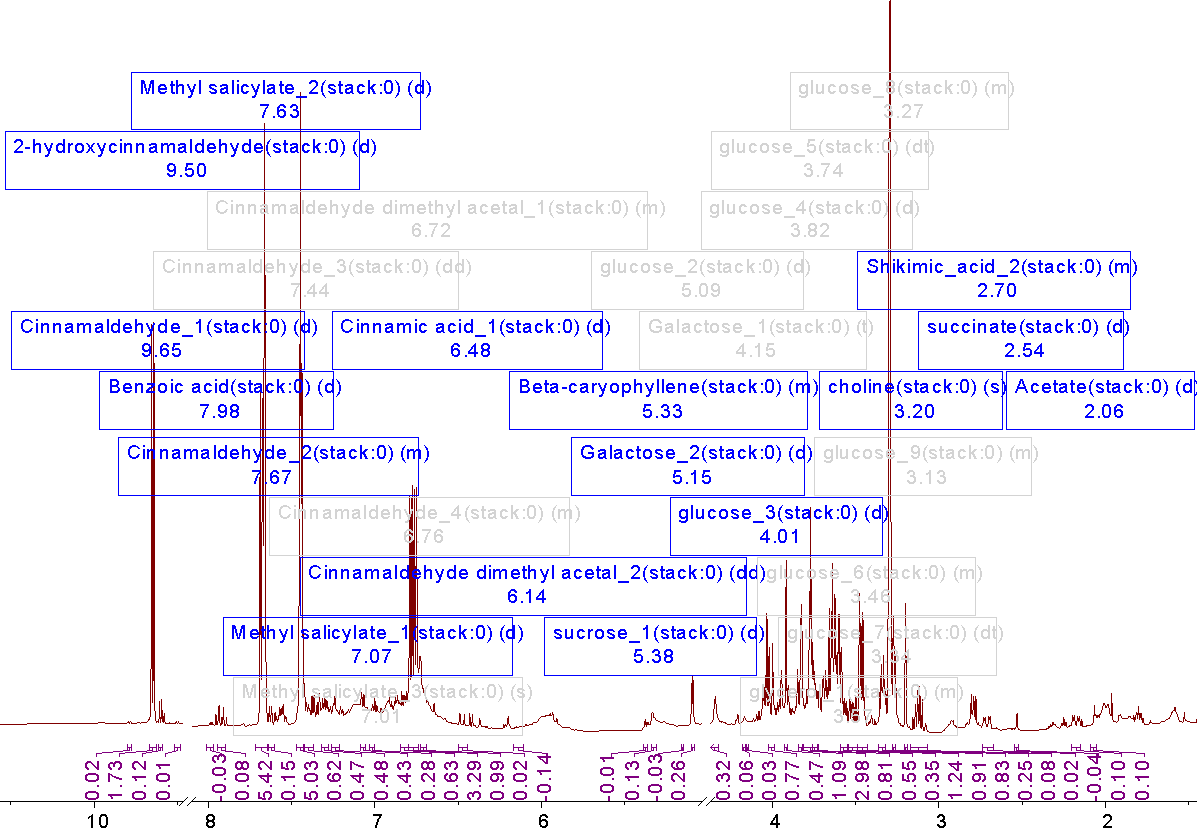


**Supplementary Figure S4.** Output of SMA plug-in of MestreNova software on a spectrum of BCE extract in CD_3_OD. Labels with the name of the molecule, the chemical shift and multiplicity are automatically reported for each signal. Signals with blue labels are used for identification and quantification of the corresponding compound, while signals with grey labels are used for identification only.


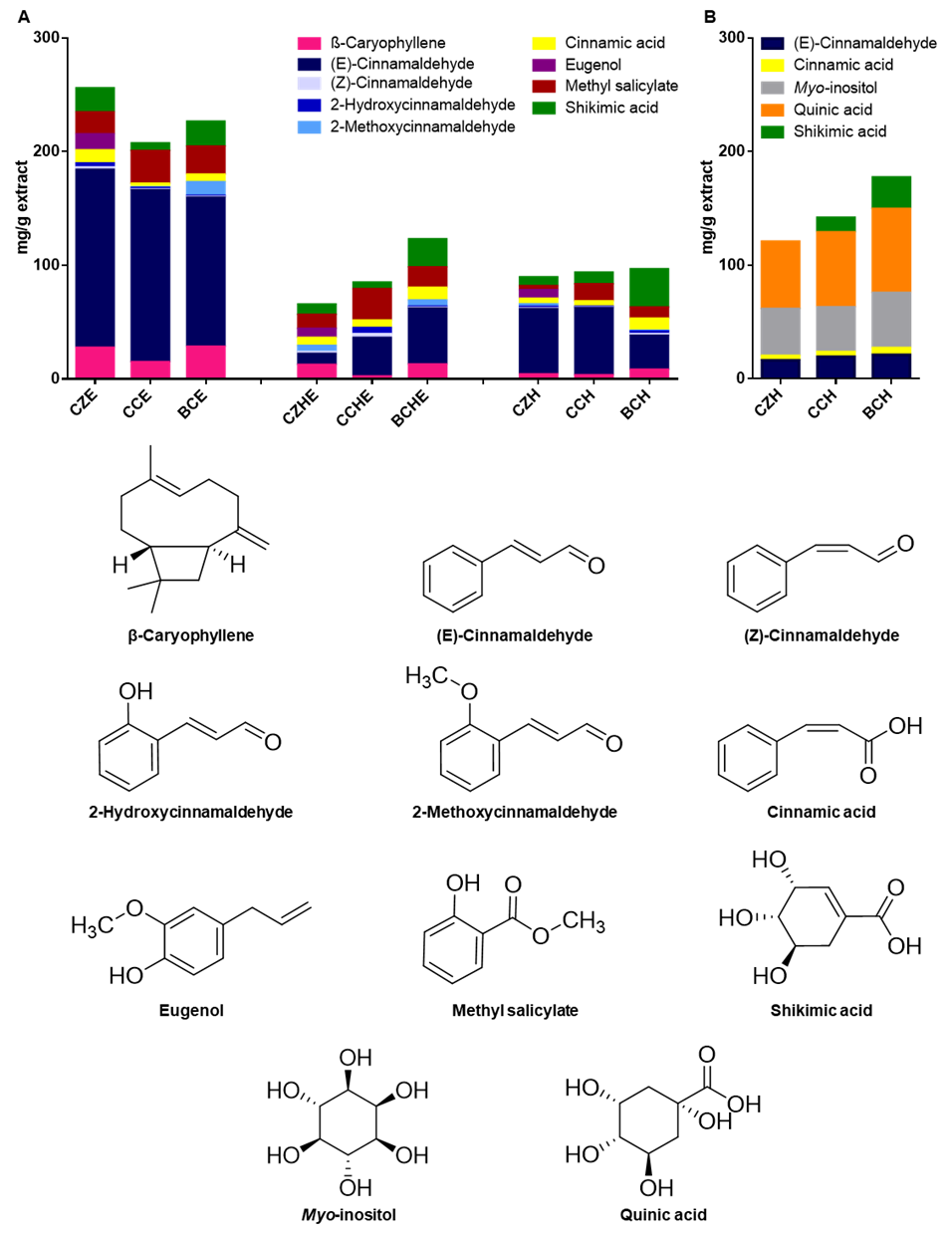


**Supplementary Figure S5**. Histograms of the sum of the concentrations of healthy metabolites in samples dissolved in CD_3_OD (**A**) and D_2_O (**B**). The chemical structures of the metabolites are also reported. BC: Cinnamomum cassia buds; CC: Cinnamomum cassia bark; CZ: Cinnamomum zeylanicum bark (CZ). E: alcoholic extraction in ethanol; HE: hydroalcoholic extraction in water (pH 4.5)/ethanol 7:3; H: aqueous extraction.


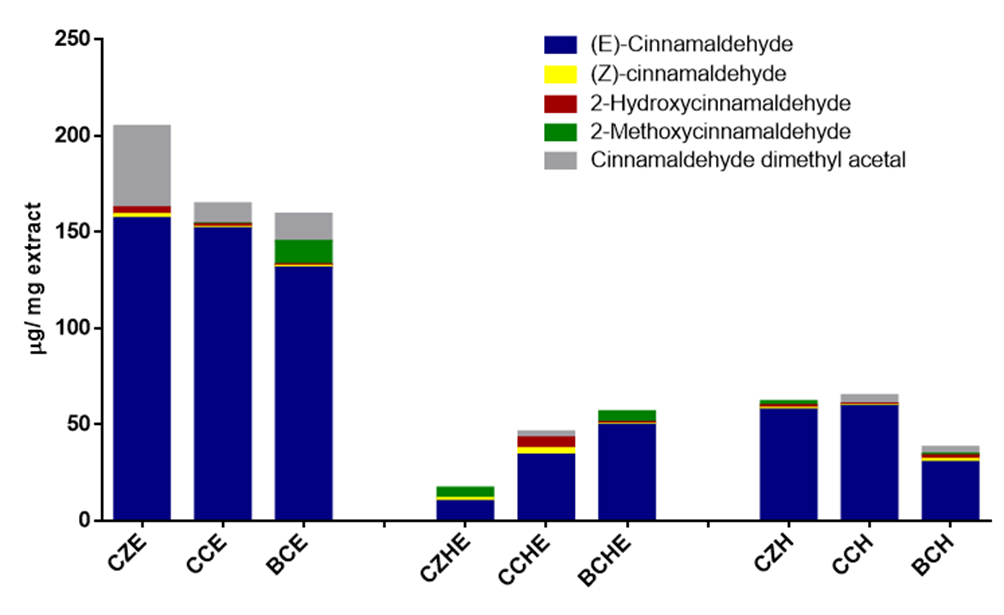


**Supplementary Figure S6.** Histograms of the sums of the concentrations of cinnamaldehyde and derivatives in samples dissolved in CD_3_OD. BC: *Cinnamomum cassia* buds; CC: *Cinnamomum cassia* bark; CZ: *Cinnamomum zeylanicum* bark (CZ). E: alcoholic extraction in ethanol; HE: hydroalcoholic extraction in water (pH 4.5)/ethanol 7:3; H: aqueous extraction.

**Supplementary Figure S7**. ^1^H-NMR spectra of the chromatographic fractions B from CCHE (**2**) and BCHE (**3**) extracts.^1^H-NMR spectra were recorded on 5 mg/mL samples dissolved in D_2_O, pH 7.4, 25 °C, 600 MHz.


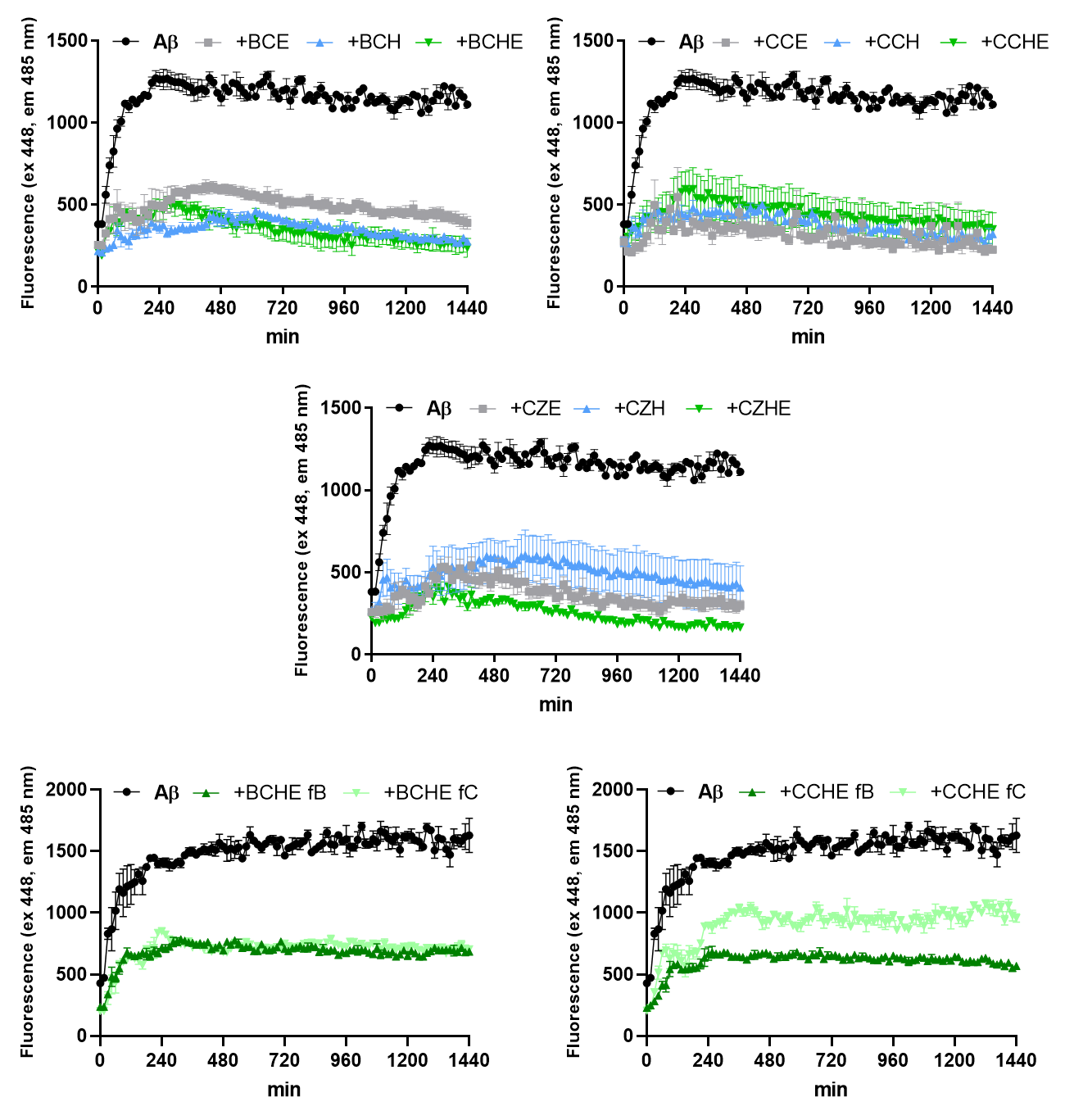


**Supplementary Figure S8**. Effects cinnamon extracts and fractions on Aβ1-42 aggregation analyzed by the ThT binding assay. Time course of co-incubation (every 15 min for 24 h at 37 °C) test compound on Aβ1-42 (2.5 μM) aggregation, determined by ThT fluorescence (excitation 448, emission 485 nm). Values are mean ± standard deviation of three replicates, after subtractions of their relative control solutions (extracts alone). BC: *Cinnamomum cassia* buds; CC: *Cinnamomum cassia* bark; CZ: *Cinnamomum zeylanicum* bark (CZ); fB: fraction B; fC: fraction C; E: alcoholic extraction in ethanol; HE: hydroalcoholic extraction in water (pH 4.5)/ethanol 7:3; H: aqueous extraction.

**Supplementary Figure S9**. **1**) ^1^H-NMR spectrum of a solution containing CCHE extract fraction B (5 mg/mL) and Aβ1-42 protein (120 μM); **2**) STD NMR spectrum of the same sample of **1**. Samples were dissolved in deuterated phosphate buffer, pH 7.4. STD spectra were acquired with 1024 scans and 2s saturation time at 600 MHz, 25 °C.
